# ‘In an otherwise limitless world, I was sure of my limit.’^†^ Experiencing Anorexia Nervosa: A phenomenological metasynthesis

**DOI:** 10.3389/fpsyt.2022.894178

**Published:** 2022-08-01

**Authors:** Emma Bryant, Phillip Aouad, Ashlea Hambleton, Stephen Touyz, Sarah Maguire

**Affiliations:** ^1^InsideOut Institute for Eating Disorders, Faculty of Medicine and Health, The University of Sydney, Camperdown, NSW, Australia; ^2^Sydney Local Health District, Sydney, NSW, Australia

**Keywords:** Anorexia Nervosa, eating disorder (ED), phenomenology, lived experience, illness experience, qualitative

## Abstract

Anorexia Nervosa (AN) has the highest mortality rate of the mental disorders, with still less than 50% of affected individuals achieving recovery. Recent calls to bring innovative, empirical research strategies to the understanding of illness and its core psychopathological features highlight the need to address significant paucity of efficacious treatment. The current study brings a phenomenological approach to this challenge, synthesizing lived experience phenomena as described by qualitative literature. Fifty-three studies published between the years 1998 and 2021 comprising a total of 1557 participants aged 12–66 suffering from AN or sub-threshold AN are included. Reciprocal and refutational analysis generated six key third-order constructs: “emotion experienced as overwhelming,” “identity,” “AN as a tool,” “internal conflict relating to Anorexia,” “interpersonal communication difficulties” and “corporeality.” Twenty-six sub-themes were identified, the most common being fear, avoidance, AN as guardian/protector, and AN as intertwined with identity. Some themes associated with current treatment models such as low self-esteem, need for social approval and feelings of fatness were less common. We highlight the significant role of intense and confusing emotion in AN, which is both rooted in and engenders amplified fear and anxiety. Restrictive eating functions to numb these feelings and withdraw an individual from a chaotic and threatening world whilst providing a sense of self around which to build an illness identity. Results have implications for therapeutic practice and overly protective weight and shape focused medical treatment models, which may serve to reinforce the disease.

## Introduction

With the highest mortality rate of any mental disorder (1–3) and a reported lifetime prevalence of up to 4% for females (4), Anorexia Nervosa (AN) presents modern-day psychiatry with a lamentable and increasingly urgent problem. Up to 15–20% of individuals will die from the illness (5, 6), with demonstrated recovery rates averaging in the region of 40–50% (7–10). Marked by excessive self-induced weight loss and refusal to maintain healthy body weight, AN is associated with numerous physical and psychological sequelae including cardiac, endocrine, and nervous system damage; life-threatening electrolyte imbalances; fertility complications; bone loss, major depressive disorders; anxiety; and suicidal ideation/attempt (11–14).

First-line medical intervention can ameliorate the physical effects of starvation (10), however the overall efficacy of existing psychological treatments is less optimistic (15). The current gold-standard treatment for adolescents, Family-Based Therapy (FBT), is successful in less than half of all cases (16, 17) and for adults, Cognitive Behavioral Therapy (CBT)/Enhanced Cognitive Behavioral Therapy (CBT-E) has been found in a recent meta-analyses to be no more effective than treatment as usual (18). Ongoing effects of poor treatment outcome are broad-reaching not only for the sufferer but for their carers and for the health system: eating disorders carry an annual estimated national economic cost of $69.7 billion in Australia and $326 billion in the United States (19, 20). When one considers that the individual with AN can be as disabled as the individual with schizophrenia (21, 22) and is four times more likely to take their own life than the individual with depression (2), there emerges a stark need to bring innovative, empirical research strategies to the understanding of illness.

Phenomenological syntheses of lived experience has been posited as one such approach (23). Increasingly, studies utilizing qualitative methods are employed to this effect, in order to assist our understanding of the social and psychological phenomena present in psychiatric illness, allowing for a rich, collaborative interpretation of illness experience (23). In AN, the individual’s psychopathological cognitions are central to the maintenance and entrenchment of maladaptive behaviors (24, 25). Shared traits and cognitions are known to exist despite variation in illness course and severity (26, 27). Perhaps the best understood of these is the egosyntonic nature of AN (28). Motivation to recover is frequently low, due to perceived benefits of restrictive eating behaviors (28). The illness has been associated with high maturity fear, intolerance of uncertainty, perfectionism, anxiety, trauma, cognitive rigidity and alexithymia (13, 26, 29–36)—however, these associations are typically measured quantitatively and thus limited to binary, researcher-derived constructs (impacting interpretive potential). Deriving a more nuanced understanding of these cognitions from the words of those who experience them may be vital to the development of novel therapeutic techniques that enhance interoception, motivation for change and self-efficacy and to understanding why existing treatments aren’t successful for many individuals.

The first to employ qualitative methods to understand the lived experience of AN was Maine in 1985 who explored the effectiveness of treatment from the perspective of 25 recovered former patients. Detailed interviews revealed few participants believed the treatment they had undergone was essential to their recovery, with many suggesting it may have exacerbated or mimicked the dynamics leading to their illness. Hsu et al. (37) again interviewed former patients about their recovery, and in 1999, Serpell published the first letter writing task in patients with AN (38), reporting that individuals felt their illness to be wholly protective; a safety blanket. From the late 90s, qualitative methods were more readily employed, however, since then have focused largely on experiences of treatment or recovery (39–43) rather than core phenomenological underpinnings of AN.

Numerous studies have examined illness experience and perception using quantitative measures, frequently the IPQ-R (Revised Illness Perception Questionnaire) (44) and measures of emotional or psychological constructs conceived by researchers. Studies using the IPQ-R have reported strong illness identity linked with chronicity and perceived low curability amongst sufferers, extreme distress as well as significant physical impairment and poor psychosocial adaptation; causal attributions of low self-esteem, emotional states and the need to be perfect (45–48). Many of these illness features are addressed within existing therapeutic models such as Schema Therapy (49, 50), Maudsley Anorexia Nervosa Treatment for Adults (MANTRA) (51, 52) and Focal Psychodynamic Therapy (53). However, current dominant treatment models— CBT, originally designed to treat depression, CBT-E and FBT—are based on the assumption that shape and weight overvaluation is the core feature of eating disorder psychopathology (54–58). Both the premise and success of treatment is frequently measured by change in Body Mass Index (BMI) and/or score on the Eating Disorder Examination Questionnaire (EDE-Q) (59), which includes items related to food and body weight/shape only and does not measure other known features of eating disorder psychopathology such as emotion dysregulation, cognitive rigidity, anxiety or delusionality (60). Given persistently low recovery rates, continued exploration of novel treatment paradigms will require us to look beyond reductive discourse focused on food and body weight concerns.

To our knowledge, no large-scale systematic review has been conducted on the AN experience as described by qualitative methodology. This review synthesizes the literature relating to phenomenological aspects of Anorexia Nervosa from multiple lenses, focusing primarily on the state of being with the disease. That is: living with it, conceptualizing it, and experiencing the cognitions and emotions that characterize the illness experience. Whilst this may encapsulate broader themes including perceived etiological and maintenance factors, relationships with others and remission or recovery, the review takes an ontological angle by examining AN as a state of being rather than focusing on peripheral aspects of living with the disease (such as treatment experiences).

The following question guided the metasynthesis: How does an individual suffering from AN experience and conceptualize their experience of the illness and its effects, particularly in relation to associated thought processes, emotions and sense of self or being? Our aim was to generate a clinically relevant, empirically informed understanding of the relationships between generated third-order concepts (defined below) and identified phenomenological sub-themes of AN, so that we may provide a rich and rigorously analyzed psychopathological framework of the sufferer’s reality.

## Methods

**FIGURE 1:**
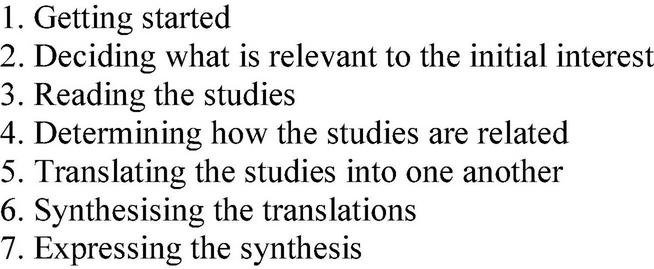
Seven steps of Noblit and Hare’s meta ethnography (62).

Methodology was guided by the ENTREQ statement (61) which standardizes the reporting and synthesis of qualitative research, and utilized a meta-ethnographic approach originally conceived by Noblit and Hare (62). This ‘line of argument’ approach generates third-order concepts using inductive qualitative analysis to express a synthesis of overarching themes and metaphors of the phenomena being investigated (63, 64). It was chosen for its interpretivist perspective, whereby rigorous reciprocal and refutational analysis of the primary studies allows for substantive theoretical innovation rather than simple aggregation of descriptive findings (65). There are seven steps to this approach (see Figure 1).

### Search strategy and selection criteria (“deciding what is relevant to the initial interest”)

A purposive, pre-planned systematic search and screening was conducted in December, 2021 according to the Preferred Reporting Items for Systematic Reviews and Meta-Analyses (PRISMA) guidelines to identify qualitative studies examining the subjective experience of AN (66). EB and PA searched SCOPUS, Medline, PsycInfo, and SSCI. Boolean operators (e.g., ‘and, ‘not’, ‘or’) were used to merge topic areas and relevant search terms related to AN (e.g., “anorexia nervosa” and “restrictive eating”), qualitative studies (e.g., “hermeneutic” and “thematic”) and subjective experience (e.g., “cognitions” and “feeling” and “experience”). The full search syntax can be found in Supplementary Appendix A. Studies from any year written in English were considered. The reference sections of included papers were scanned for further published studies that might meet inclusion criteria. Purposive (rather than exhaustive) sampling was chosen by the researchers due to the broad nature of the concept under investigation and the potential volume of related studies, as well as the interpretive (rather than predictive) nature of qualitative syntheses, which may be better suited to purposive sampling strategies (67, 68).

Included studies investigated AN or related restrictive eating disorders, in any age group or gender; were qualitative (or mixed-methods) in design (including but not limited to semi-structured and focus group interview studies; journal, diary or letter-writing tasks; narrative studies; case studies or vignettes); examined *either* as primary or secondary aim phenomenological aspects of the cognitions, emotions or embodied experiences of AN; and were peer-reviewed. Excluded were ‘gray’ literature such as chapters and books, Masters’ theses or dissertations; and studies exploring experiences of recovery, treatment, relationships with treating professionals or any aspect of AN not directly about the internal experience of the condition.

### Quality appraisal (“reading the studies”)

The CASP qualitative checklist [Critical Appraisal Skills Program (69)] was used for quality assessment of included studies. The 10-item checklist assesses a study’s statement of aim; appropriateness of methodology and research design; efficacy and appropriateness of recruitment strategy and data collection; ethical issues in the research process; rigor of data analysis; coherence and transparency of findings; and overall value. We used a 3-point quality rating for each item on the checklist (70), where 1 is poor quality, 2 moderate quality and 3 high quality. This yielded a total maximum possible score of 30, with scores closer to 30 indicating higher quality. EB independently appraised all 53 studies. A second and third reviewer (PA and AH) independently rated 24 studies (45%) and 5 studies (10%) each. Inter-rater reliability was calculated as a percentage agreement. Raters assigned the same score to 24 (83%) of the 29 studies. All disagreements were discussed and resolved by EB and PA in order to arrive at a final appraisal for the remaining five studies.

### Data extraction (“reading the studies”)

A data extraction table was developed for the purpose of this study and recorded the following information: Author, Year, Country; Title; Aim; Recruitment setting; Participant demographic characteristics; Sample size; Qualitative methodology; Results; Discussion; Summary Findings. Data from each study relating to the subjective experience of AN (specifically, cognitions, emotions, beliefs and the structures of consciousness underpinning and maintaining the anorexic mindset) reported under ‘Results’ or ‘Discussion’ were read twice by the first author and once by the second author.

### Analysis and derivation of themes (“determining how the studies are related”)

All extracted data was analyzed and codified line-by-line by the first author for related and recurring first order (lived experience or participant-reported data) and second order (primary study authors interpretation of these) constructs. Key words were recorded alongside the data extraction table (e.g., ‘sadness,’ ‘overwhelming,’ ‘anger’) and exhaustively grouped into descriptive themes, which were reviewed by and discussed with the second author. Any theme identified across three or more papers was categorized as a ‘sub-theme’ for the purposes of the metasynthesis.

### Writing the synthesis (“translating the studies into one another,” “synthesizing the translations,” and “expressing the synthesis”)

All extracted data and key words were translated reciprocally and refutationally by the first author in an iterative and categorical data saturation process, whereby the translation continues until no new themes emerge (71). A reciprocal translation identifies shared themes by juxtaposing first and second order constructs between studies. This generates new concepts which provide a theoretically rich account of the given phenomenon (65). A refutational translation aims to understand and reconcile contradictory themes within and across the studies (65). This process generated overarching third-order constructs which were discussed with and agreed upon by the other authors. At the completion of the process, the first author wrote the synthesis (discussion) as an expression of the overarching themes present in the studied phenomena. All authors read and agreed upon the synthesis.

## Reflexivity statement

The authors acknowledge their own implicit biases in approaching the synthesis. All authors self-identify as Caucasian, upper middle-class eating disorder researchers and academics, working under a biomedical framework and within a universal healthcare system. The first author has lived experience of AN. The second author has been a carer of an individual with AN. The third, fourth, and fifth authors are clinical psychologists and researchers who have treated individuals with AN and studied the illness for some years.

## Results

**FIGURE 2:**
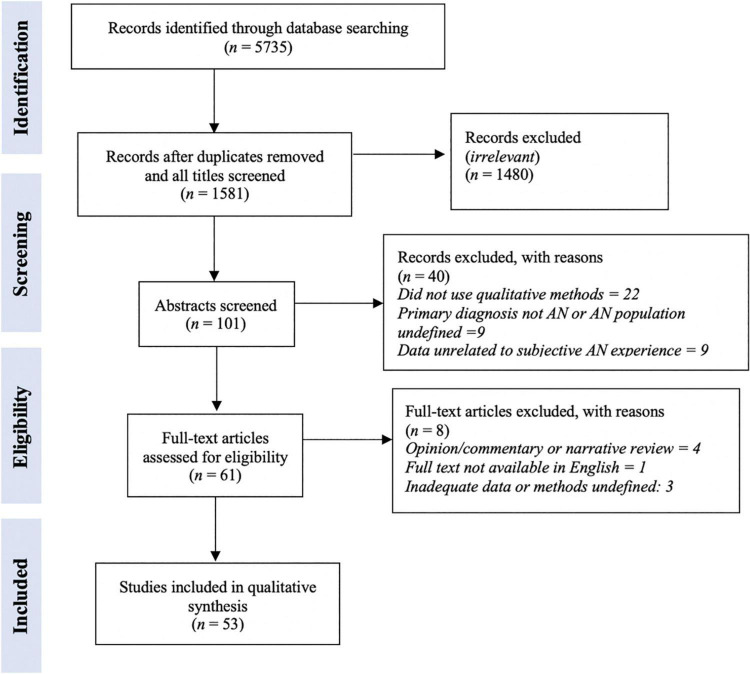
PRISMA flow diagram of included studies.

Database searching identified 5735 records, 1581 after duplicates were removed. Initial title screening identified 101 studies for abstract screening, from which 40 studies were removed. A total of 61 studies went to full text review and from this reading, 8 were excluded for reasons outlined in Figure 2.

Fifty three studies were included in the analysis, totaling 1557 participants aged between 12 and 66 suffering from AN or subthreshold AN (Supplementary Appendix B). All identified studies were published between the years 1998 and 2021. Only three studies were determined to be of low quality (<20) – these were not excluded, but CASP results were given due thought when conducting the synthesis (Supplementary Appendix C).

Included studies were heterogenous, varied in setting, sample size, stage of illness, methodological design, data extraction and analysis. 49 studies were conducted in high-income countries (72), most commonly the United Kingdom (*n* = 31 studies), with well-established, universal health-care systems (19). Recruitment took place in a wide range of settings, including specialist eating disorder inpatient units (current patients *n* = 14 studies; former patients *n* = 3 studies), outpatient services (*n* = 26 studies), community mental health (*n* = 7 studies), and through traditional (*n* = 5 studies) and online (*n* = 11 studies) advertising. While each study explored a multitude of aspects of the lived experience hence their inclusion, the primary aims were as follows: 10 papers described emotions associated with AN; 25 papers explored meanings attributed to anorectic behaviors and self-starvation; 6 looked at experiences of the body or fatness and 10 examined perceived biopsychosocial causes and effects of the illness.

We identified six key third-order constructs in the analysis, and twenty-six sub-themes. In order of the strength and quality of evidence in addition to the frequency with which they were mentioned, the third order constructs were “Emotion as overwhelming,” “Identity,” “AN as a tool,” “Internal conflict relating to Anorexia,” “Interpersonal communication difficulties” and “Corporeality” (see Table 1). The most frequently identified sub-themes were Fear (*n* = 26 studies), Avoidance/Numbing (*n* = 26 studies), Guardian/Protector (*n* = 24 studies), Control (*n* = 23 studies), Achievement/Strength (*n* = 22 studies), and Role/Intertwined (*n* = 19 studies) (Table 2). Studies are categorized by third-order construct and sub-theme in Supplementary Appendix D. Example quotes for each construct are provided in Table 3.

**TABLE 1:** Identified third-order constructs and sub-themes*.

**(1) Emotion as overwhelming**	
82 statements	Fear (26)
	Sadness (16)
	Loneliness/isolation (12)
	Difficulty understanding or identifying emotions (10)
	Shame (7)
	Hopelessness (6)
	Anger (5)
**(2) Identity**	
79 statements	Achievement/strength (22) Role/intertwined (19) Low self-efficacy/self-esteem (15) Perfect/pride (12) Lost sense of self (11)
**(3) AN as a tool**	
76 statements	Avoidance/numbing (26) Guardian/protector (24) Control (23) Becoming needless/self-punishment (3)
**(4) Internal conflict relating to Anorexia**	
30 statements	In control vs. out of control (12)
	Functional impairment (9)
	Wish to maintain illness vs. wish to recover (9)
**(5) Interpersonal communication difficulties**	
24 statements	Difficulty verbalizing distress (10)
	Difficulty with interpersonal relationships (8)
	Need for social approval (6)
**6. Corporeality**	
20 statements	Need to disappear (7)
	Disconnection/incongruence with bodily reality (7)
	Hunger experience (3)
	Need to be empty (3)

*References by theme and third-order construct available in Supplementary Appendix D.

**TABLE 2:** Top 10 sub-themes.

(1) Fear
**=1.** Avoidance/numbing
**(2)** Guardian/protector
**(3)** Control
**(4)** Achievement/strength
**(5)** Role/intertwined
**(6)** Sadness
**(7)** Low self-efficacy/self-esteem
**=8.** Perfect/pride
**=8.** In control vs. out of control
**=8.** Loneliness/isolation
**(9)** Lost sense of self = **10.** Difficulty understanding or identifying emotions = **10.** Difficulty verbalizing distress

**TABLE 3:** Reflective quotes by identified third-order construct and sub-theme.

	Example Quote
**(1) Emotion as overwhelming**	
Fear Sadness Loneliness/isolation Difficulty understanding or identifying emotions Shame Hopelessness Anger	“He helped me to see that whereas it feels like it’s food, most of the time that what I’m afraid of is life itself. I’m just so frightened of it, because of the hurt that went on in my childhood.” (101) ‘It helps me cope with life and keeps me safe and safe from the badness in the world…Keeps my demons locked away, of inadequacy, failure, weakness, indecision, reaching expectations, criticism, feeling vulnerable, exposed, angry, sad etc., easier to avoid situations where I feel those feelings. Life is less complicated, less fearful, less uncertain.’ (77) ‘When I feel sad, I’m actually a very sensitive person and I often feel sad and begin to cry, then I always react to food—I don’t eat or I throw up.’ (122) ‘I feel sad. And when I am sad, I feel burdened and heavy… and then comes the urge to lose weight.’ (105) ‘My life is a mess. I am desperate and depressed. I really believe that I am extremely sad. But then I immediately get the thought: what do I have to be sad about. After all, I should be fine.’ (73) ‘…and I was tired all the time and cold… thoroughly miserable. You think of food all the time… it’s horrible.’ (123) ‘I don’t belong in the world. I’ll never be good enough for your world and I most certainly don’t belong in the real world, I just don’t belong.’ (77) “The anorexia has taken me out of the world all alone and into its own world where there is no one but me…I now think I don’t have any close friends because they all got fed up with me”; “I see life going on around me but feel distanced, displaced from it.” (77) ‘I haven’t cried since I was 13 years old… Sometimes I wish that I could sit down and cry and let my feelings out. But they’re actually gone. I really can’t recognize them. I don’t know if I’m sad or angry or what I feel…’ (122) ‘I’m not very good at being aware of my emotions or describing my emotions or probably responding to negative emotions […] sometimes complicated difficult emotions get condensed in my mind into ‘I feel fat.’ (121) ‘Shame: it is the life nerve… it is somehow one of the two or three motivating or constantly present moods I have or have had in my life, as long as I can remember and for everything… I have always been a super ambitious person. But I am ashamed of not being able to live up to them, not being able to take hold of them, not to have this masculine strength for just going for it and accomplishing it. But then I also feel ashamed of having these ambitions…’ (73) ‘I am ashamed of playing the hypocrite; using vomit to be thin is not strong and firm self-control, but it is cheating… I am preoccupied with being thin, but then I am also preoccupied with the fact that this is stupid vanity…’ (73) ‘When I felt guilty, I exercised a lot and vomited… I cried, I felt bad and the next day I didn’t eat (II). Everything I felt was transferred to food, and I stopped eating (VI).’ – (89) ‘… I feel ashamed about everything. I feel ashamed about feeling ashamed.’ (73) ‘I feel like really, really hopeless and it’s like soul destroying. Because I can’t see anything changing.’ (124) ‘… just like, I suppose that I felt like they had given up on me so I gave up on getting involved, just like I can’t be changed so what’s the point really.’ (102). ‘Now when I’m supposed to eat more food, I’ve noticed that all my feelings are surfacing. And then I see that I actually have a lot of anger. ‘Cause when I eat I always become angry and irritated. It’s like a nasty restlessness inside me. It feels like an evil energy. I don’t like becoming conscious about it.’ (122)
**(2) Identity**	
Achievement/strength Role/intertwined Low self-efficacy/self-esteem Perfect/pride	“Feel a sense of purpose, a drive”; “Sense of achievement/superiority”; “Challenges such as walking, exercising, restricting provide a sense of achievement, structure and routines occupies my mind. Make me feel strong and push myself to extremes, denying myself things.’ (77) ‘…very focused: if I do something I do it 100% and I won’t give up. I like proving that I can be successful, I’ve always been like that, even as a child… I was always really serious about my schoolwork and really serious about achieving my goals so um, when I want something really badly, I make it an obsession, so it becomes my life.’ (125) ‘I am the anorexia. Nothing else but anorexia… it’s like if I give up that name what else is there?’ (116) ‘At the moment, anorexia plays a massive role in my life. Everything I do or think, I relate it to anorexia in some way… They [anorexic behaviors] are everything to me, almost my entire life…My anorexia is my life. It is who I am’ (95) ‘I was too busy concentrating on being anorexic at school. I was too busy on saying the right thing, doing the right thing. I truly believed that my role in this world, my place, was to be the anorexic. Because you have the dominants, the leaders, the thinkers, I was just the anorexic, that was who I was.’ (92) ‘I am a hopeless person, not worth loving. Everything I do is stupid. I should not have been born, and very often I do think that I do not deserve to live. I cannot stand myself…I take up space, too much space; there is too much of myself. I can also notice it in the others at school. I realize that it is better to be worn out and small. Then I don’t take up more room than I deserve.’(73) ‘It just made me feel really powerful…my legs would feel like jelly…but you push yourself to keep going, […] it’s not necessarily pleasant, but it still gives you a buzz, a sense of pride…’ (126) ‘One of the sort of big aspects of my personality is perfectionism and I think that plays a really big role because it’s the kind of perfectionism that where everything is constantly measured against standards so at school it was fine because you can measure yourself by expecting yourself to come top in everything and that’s really clear and then I think perhaps one of the reasons why the food stuff took over was when I’d finished school because there aren’t all those things that you can measure yourself against’ (97) ‘I felt like a sense of pride that I’d managed to get my BMI lower than anybody else in this unit […] it was like, you’d gone as far as you could go kind of thing, it feels like on top of the world.’ (126) “I am very sick, and I have destroyed myself. But I am also proud because, in my own way, I keep going and don’t give in.” (73)
Lost sense of self	‘It doesn’t feel like me anymore, I feel like I’ve lost myself.’(80) ‘When I was so ill I felt like I was two people. I’d got the anorexia and I’d got me and I was really confused and it was a battle.’ (101) ‘[AN is…] something that’s me, that’s mine. It’s a way of life. And it was like, well it was like me. It’s like a way to have an identity… And if I didn’t have it, if I wasn’t thin… then I wouldn’t have an identity. I’d just be this big bad blob… Before I’d just felt like nothing. Now I had something to focus on and something to be.’ (116)
**(3) AN as tool**	
Avoidance/numbing Guardian/ protector Control Becoming needless/self-punishment	‘You close my eyes when I don’t want to know and knock me out when I don’t wish to feel.’ (77) ‘Food was all I could think about. I used it to comfort myself and run away from my life….[F]or the most part, it STOPS me feeling. It numbs out pain, fear, anger, rejection. Takes away anything/everything except it in itself […].’ (90) ‘(AN was) my thing that I could turn to that would like numb all sadness or anger.’ (124) ‘My anorexia was there when everything else seemed unpredictable, excessive, in a frantic state. Its austerity, its plain, straightforward and concrete nature infused the unsure with something safe.’ (105) ‘It’s like a protective thing and it feels like… round my heart.’ (96) ‘In an otherwise limitless world, I was sure of my limit.’ (105) “To me the world is out of control and messy and horrific and that’s why, I wasn’t always aware of this, but that’s why I’ve lived more than half my life in institutions because it reduces dramatically the amount of uncertainty that I’m faced with and chaos.”(88) ‘I really need some fixed points in my life. I need to feel my skeleton. I want physical contact with my bones.” (Hanna). “When I don’t have access (to bones and skeleton), when there is something between what I feel when I touch myself and my inside, then I get scared. I don’t like it. Things are blurred. … I want to be hard.’ (105) “Every person in the whole world deserves to be happy and enjoy good health… except me. It’s an awful feeling.” (99) ‘At various points in their anorexia participants described not allowing themselves to have showers, to spend money on themselves, to be “warm, or… comfort or… being allowed to sit on a soft chair” (Jane). Sarah similarly would not allow herself to be warm, to receive gifts, to use self-care products and for a period insisted “if I was going to be nourished the only nutrition I would allow was an NG [nasogastric] feeding.”’ (107)
**(4) Internal conflict relating to Anorexia**	
In control vs. out of control Functional impairment Wish to maintain illness vs. wish to recover	‘I’m that much in control that I’m out of control’. (127) ‘I believed that I had control over food and the eating disorder but the question is whether that’s really the case. It’s rather the eating disorder that’s in control.’ (99) ‘It’s like you’re slipping into a whole different world… like you’re stepping out of your body, you’re looking at yourself and you’re doing these things and like even though I felt I had control over it there were times when I really didn’t think I had any control of it at all.’ (80) ‘[I would] rather be HIV-positive than have this life.’ (100) ‘You’re erasing my memory as well as my mind - suspending my aspirations, muddling my wishes and interfering with my spirit. You’re sucking me out. You quash my dreams…. The hate sometimes is excruciating like a pain you want to leave, only this desire and control slots itself into every corner of my mind and I have allowed it to run parallel with my everyday life.’ (77) ‘I lost my job, I had to resign in March because I wasn’t well enough to go back. I couldn’t do my duties- I couldn’t even, my uniform weighs a stone, I couldn’t even wear my uniform so I had to give that up and because I had to give that up I had to lose my flat, I couldn’t afford the mortgage on that and I’ve just gone bankrupt because my flat was in negative equity and I’m living with my parents now. I have lost everything through the eating disorder absolutely everything.’ (97) ‘It took over completely and I had nothing left. All my friends were bored of me…I missed lectures and loads of work. And I was tired all the time and cold… thoroughly miserable… You think of food all the time… it’s horrible.’ (123) “I still have my eating disorder because I know what to expect from it and it is something that I can rely on. I stay with my ED like an abused woman stays with her abuser. It is truly a love-hate relationship that I cannot escape.” (90) ‘I don’t want to die, but I don’t want to recover, though-not yet… mostly because of fear, and a small bit of me wants to get better sometimes, but I wouldn’t know how.’ (95) ‘I don’t understand why I can’t let it go.’ (91)
**(5) Interpersonal communication difficulties**	
Difficulty verbalizing distress Difficulty with interpersonal relationships Need for social approval	‘I don’t let anybody see how I feel. I hide it. If I’m sad, I’ll do anything to prevent others from seeing it. I live in my own little world. Nobody knows how bad I feel. I just pretend to be glad, but as soon as I come home that’s not how I feel.’ (122) ‘Well, it’s what I saw, if you express your anger that’s bad, you’re naughty. If you cry, you’re a fool, you’re silly, cos everybody else doesn’t cry, it’s wrong…’ (78) ‘It’s my way of talking to the world. It tells everyone what I can’t. It allows me to show them how much I am hurting, how scared I am, how much I feel I am without’. (95) ‘I just felt like… I was completely on my own and no-one felt the same way as I did (yeah) and that nobody understood how I was feeling.’ (113) ‘ ‘I’ve never had a boyfriend, although I would like one… But although I say that… I don’t if I could allow myself to have that affection or allow myself to – allow someone else to get close to me.’ (114) ‘… I’ve always been a go-between… trying keep peace with everybody. I’ve never really spoken up in my life until recently. Erm, I go from one to the other, agreeing with that person, agreeing, and then going back with the other members of the family and trying to agree with everyone just to keep everybody happy.’ (114) ‘Once in a while if I go out to meet people, I almost panic! I get so paranoid! As soon as I get to the meeting place, I feel very uncomfortable. And then it’s like I immediately start focusing on my body, ‘Oh my god, I can’t even feel my hipbone. I should have put something different, because then I wouldn’t look so fat’.’ (115)
**(6) Corporeality**	
Need to disappear Disconnection from/incongruence with bodily reality Hunger experience Need to be ‘empty’	“I was not able to limit myself; I did not know where I started and where I ended. That is why I did like this: (She describes with her whole body how she diminished herself). Like from a grape to a raisin.” (105) “Oh, it was a little bit like not being here. Always, like not wanting to be here, here on Earth, like not being connected, because food connects you. It connects you with others, with the Earth. Not wanting to eat before was like not being here, like being and not being. Like flirting with life all the time, eating and vomiting, being—not being, you know? (XIII).” (89) ‘There’s sort of a feeling there of wanting to sort of just fade into the background literally.’ (116) ‘Just let go and hope you disappear before you hit the bottom.’ (117) ‘I remember sort of…looking in the mirror and actually being surprised that I saw a form in the mirror and not just nothingness.’ (116) ‘I didn’t look at myself in the mirror, I stopped existing [….]… this is what’s really getting in the way of really being… I wished I were foam and I could float, and say goodbye to my body, I didn’t want it at all, I didn’t want anything to do with it.’ (89) ‘They’re both a high because… if you’ve been restricting yourself and you binge, you do get a high…you know blood sugar high and you can actually feel it, it makes you feel dizzy…and then if you, umm if you restrict you get the same sort of high which is a light, it’s different but it’s the same in the sense that it’s light headed, makes you feel dizzy because you haven’t had enough food.’ (80) ‘… It started somehow when I began using food as dope, and this I was ashamed of, and then I began using hunger as dope, I am like a drug addict with my ‘uppers’ and ‘downers’…’ (73) ‘I am so confused. It is simply too much for me. I have to reduce. I am completely filled up. In some way or another I do have to empty myself.’ (105)

### Emotion as overwhelming

Anorexia Nervosa was perceived to be both a consequence and cause of profound negative and frequently unmanageable emotion, which was the dominant theme of the studies. Participants described acute levels of distress they were unsure how to respond to, or which confused them. The distress was frequently suppressed for fear of rejection from others if outwardly emotional and/or an inability to verbalize confusing feelings. In some cases, this was linked with traumatic childhood experiences, but in others without obvious cause. There was a desire to completely ‘extinguish’ the body, or the ‘vessel’ of emotion and when individuals felt threatened by their emotions, their unconscious reaction was to evaluate their appearance negatively. This was particularly salient in response to fear and sadness. Within this construct, seven sub-themes were identified: fear, sadness, loneliness/isolation, difficulty understanding or identifying emotion, shame, hopelessness, and anger.

The overwhelming emotion described had a visceral quality and was predominantly identified as fear or anxiety. Fear was a core feature of the AN experience in half of all studies (*n* = 26). Participants frequently recalled being anxious children and continued to view the world as a frightening, chaotic or threatening place. For some, childhood experiences of bullying, abandonment, sexual or emotional abuse and bereavement marked clear developmental pathways of fear conditioning. The fear was ‘ever-present’ and typically associated with uncertainty. It could be linked to strong physical sensations such as a lump in the throat or extreme restlessness and was often a response to the illness itself: participants were intensely afraid of gaining weight or ‘losing control,’ and—paradoxically—never getting better. Participants described restricting food to suppress fear and purging food to release it.

The second dominant emotion described was sadness (*n* = 16 studies), which was strongly linked to feelings of heaviness or fatness and body dissatisfaction. Like fear, it was frequently and deliberately inhibited. Participants ‘masked’ sadness in front of others so as to protect them from perceived burden and not to appear ‘weak.’ Some felt guilty for having ‘nothing to feel sad about.’ One author interpreted the latter as the shame of being self-pitying which was expressed concretely through self-denial of food (73). Contrarily to fear, sadness appeared to be a consequence of the eating disorder more often than it was causative. It was a more acceptable emotion to release when alone than were other emotions such as anger, which were perpetually inhibited. Sadness can be seen as a consequence of the loss of something valued, failure to achieve a goal, or sense of lack of control (74), so it is unsurprising this emotion was pervasive and associated with strong feelings of hopelessness. Again, restrictive and purging behaviors regulated and stifled these feelings. In one case, AN was described as a deliberate but less overt method of suicide.

The next sub-theme was loneliness and isolation (*n* = 12 studies). Research has consistently linked AN with significant socio-relational impairments and attentional biases to social threat (26, 75, 76) however there was conflict between studies as to whether these impairments precede AN or are a consequence of starvation (or both). AN typically protected individuals from loneliness, by providing them with a sense of companionship, whilst paradoxically—particularly in the later stages of the illness—exacerbating it. Feeling ‘left out’ of or deliberately avoiding normal peer events and receiving negative feedback about the illness (e.g., family members or friends blaming the person for being ill or being told to ‘snap out of it’) increased feelings of loneliness and isolation. Many of the individuals in these studies had stopped being invited to events; some had acrimonious relationships with parents; and most had experienced some degree of criticism from either family or peers at a young age that caused them to withdraw socially. Study authors thus observed that the discrepancy between communication and social skills scores in individuals with AN may be better explained by interpersonal avoidance than skill deficit, driving isolation in a way that results in profound solitude and self-obsession (77).

Several of the present studies substantiated AN’s association with alexithymia (*n* = 10). Participants described immense difficulties understanding or identifying emotions and an inability to distinguish between physical sensation and cognition (e.g., feeling ‘fat’ vs. feeling sad). This may have been related to poor meta-emotional skill modeling received growing up for some individuals (who had frequently experienced or witnessed trauma leading to intense feelings of anger and sadness) who were not taught to regulate or express their emotions. This resulted in “emotional confusion, overcontrol of emotions and the use of eating disorder to express emotions” [(78), p.294]. Feeling emotional translated to ‘feeling fat’ for many of those participants who had difficulty identifying emotion. Participants in one study alluded to increased emotion suppression at lower body weight, with authors suggesting alexithymia in AN may be due to “compromised cognitive functioning [rather] than an implicit difficulty with identifying emotions.” [(78), p. 299, (79)].

A deep sense of shame, interpreted by some study authors as a maladaptive dysregulation of self-esteem, was described by individuals in seven studies. Rance et al. (80) identified two types of shame in AN, which were reflected across the studies: ‘globalized internal shame’—that is, general feelings of inadequacy and humiliation; and ‘focused shame’—the shame of having the illness (80). Shame has been regarded as an adaptive response which causes us to withdraw from social rejection and possible violations of self (81, 82). Internalized shame is common in AN (83, 84) and was in some studies interpreted as a response to objective experiences of violation, abandonment or humiliation as a child; and in others came from heightened trait sensitivity to social cues of attractiveness and success (73, 80, 85, 86). Participants spoke of intense self-loathing and self-disgust, particularly regarding their body image. Here, individuals described shame relating to low self-worth, focused shame, and a paradoxical shame that they were not more ill (and thus were ‘failing’ at AN). Focused shame was most frequently broached, with participants deeply aware of the ‘irrationality’ of their illness and the control it had over them. They were acutely conscious of the stigma associated with their illness and felt embarrassed that they could not do something as ‘basic’ as feed themselves.

Hopelessness was described in 6 studies. Individuals were more likely to report feeling hopeless if they had experienced a chronic/longstanding course of AN and/or had received extensive treatment intervention to little effect. Some reflected on experiences of being ‘passed around’ services and of feeling abandoned by healthcare professionals. Negative feedback from staff was common, particularly in medical wards and non-eating disorder services, amplifying feelings of isolation and despondency. The ill-effects of the illness, particularly in its chronic form, were apparent and included insomnia, extreme irritability, constantly feeling cold, being unable to control weight loss beyond a certain point and experiencing a multitude of physical problems. As a consequence, participants frequently desired full recovery but the chronicity and/or perceived strength of the illness meant they despaired of their ability to achieve it, the very idea of recovery a fantasy.

Anger was mentioned infrequently when compared with other negative emotions (*n* = 5 studies), however, was dynamic and directed threefold: at the world, the self and the illness. It was one of the most difficult emotions for individuals to process and appeared to be the most disliked. This was seen by study authors to be linked to its uncontrollability and unpredictability. Several participants spoke of vomiting, exercising and overt self-harm behaviors to release their anger, which was pent up inside. This anger was sometimes directed at others (though never in their presence – with most of the individuals being highly conflict avoidant and suppressing anger to protect interpersonal relationships) but mostly at the self, which reflects research suggesting individuals with AN deflect their anger to their bodies in the form of self-disgust (87). Anger was particularly potent on admission to inpatient treatment when eating disorder behaviors were restricted and could not be used to suppress emotions. Participants described intense anger when having eaten or having food in their stomach. Fox observed strong links between feelings of anger and purging behavior, with individuals who vomited frequently describing childhoods marked by fluctuations between episodes of anger and emotion-denial (78).

### Identity

The second third-order construct established was identity. AN was described in almost every study as in some way attached to an individual’s identity and sense of self: a role one felt compelled to play out, a sole purpose in life, or inextricably linked with personality. It was repeatedly asserted that the restrictive eating originally used to boost confidence and ameliorate underlying anxieties eventually became an identity in and of itself (88–90) particularly as it became more entrenched and after frequent contact with healthcare services (91).

The first sub-theme was achievement and strength (*n* = 22 studies). This concept refers to the feeling of exceptionalism gained from being able to do something (starve) that “not many people [can do]” [(92), p. 298]. This sense of achievement and mental strength peaked in the early stages of the illness and was reinforced when positive external feedback was received in response to weight loss. Individuals were typically high-achieving perfectionists in other areas of their lives and losing weight was simply another thing to be good at. They appeared to desire difference from others, if this difference was seen to be positive. This was understood to play out on a backdrop of insecurity and low self-esteem. Marzola delineated three distinct types of AN: the ‘need for excellence’ group, the ‘safe-seeking’ group and the ‘entrenched AN’ group (85). 91% of the first group identified difference (achievement) as a positive. In Freedman’s thematic letter analysis (where participants were asked to write two letters to their eating disorder, one addressing it as a friend and the other as an enemy) 47% of adults with AN mentioned themes around difference (93). O’Connell et al. (94) found feelings of exceptionalism played out slightly differently – in the case of that study, participants believed their bodies were exceptional or different from everybody else physiologically. This meant they needed less food than a ‘normal’ person to maintain the same weight, and thus were obliged to engage in disordered eating behaviors (94).

AN was strongly intertwined with an individual’s sense of identity (*n* = 19 studies). This was a major barrier to recovery and became more profound the more enduring the illness (95). Participants describe needing to fulfill the anorectic role—both for their own self-efficacy and to match perceived expectations of others: “I’m the girl with the eating disorder, so I’m the one who should be like, thin” [(96), p. 221]. This identity went so far as to give participants a ‘persona’, a self-image. It filled an emptiness or void, whereby participants had lost their sense of self, or were in a state of ‘becoming’ (frequently the juncture between childhood and adolescence) when they first got ill. Many participants spoke of ‘being’ anorexia: of not knowing who they were without it, and of having had it for so long they wouldn’t know who they were if they recovered. This was self-perpetuating, as one participant described: “I can’t work because of Anorexia and low mood [….] – when people ask you what you do, well not a lot really… I don’t really do a lot and so it’s almost like that allows the illness to become stronger because you don’t have another role.” [(97), p. 133]. Participants felt AN filled a gap in their lives caused by a failure to achieve valued roles or purpose. Recovery, then, was a threat to the self. Without it, individuals would be “nothing,” “insignificant” or “unreal” [(96), p. 221]; thus the illness has distinctive effects on role formation (98).

Low self-esteem was related to shame, however, as a standalone concept was not addressed in the studies as frequently as might be expected in this disorder (*n* = 15 studies). Some participants described an underlying chronic low sense of self-efficacy and self-worth, which was usually accompanied by hostile self-talk (“not worth loving,” “stupid,” “do not deserve to live”) [(73), p. 86, (99)]. This perpetuated eating disorder behavior as individuals sought to ameliorate their perceived failures and/or atone for being a generally ‘bad’ person. Participants appear to have internalized messages around fatness and badness, with feelings of fatness leading to feelings of incompetency, laziness, or being a ‘bad person’ who had somehow failed.

Participants were typically highly perfectionistic and expressed pride relating to the AN not independently of shame (*n* = 12 studies). This was experienced in a multifactorial way: perfectionism as both trait and causative in the development of AN; and associated sense of pride as maintainer of anorectic behavior. Some participants failed to see that the ‘bad’ parts of AN outweighed the ‘good’, instead committed to an idealized AN marked by “enviable, ethereal delight in emaciation” (100). Pride in ability to maintain a low weight diminished as individuals became more chronic, and many struggled with nostalgia for the earlier stages of their illness when they still felt pride. Some described being frustrated that they had ultimately been unable to ‘perfect’ AN (whether they had stopped being able to lose weight easily, been hospitalized or became aware that their behavior was pathological). Surgenor et al. observed that contrary to general belief, the individuals in their study were not in fact committed to therapy in an attempt to defeat AN but rather in an attempt to perfect it (i.e., wanting to ‘fix’ the psychopathology but not the weight-loss) (100). One participant described leaving school as a turning point in her AN: as there were no longer any tests or standards against which to measure herself, her perfectionistic obsession with achievement was driven into weight-loss. Similarly, participants had a tendency to pursue discipline and self-control in an extreme and perseverative way. Values seen to be virtuous were pursued to the detriment of all else, and most spoke of nothing ever being ‘good enough’.

Finally, the split between the anorectic self and the ‘real’ or ‘authentic’ self was frequently experienced as confusing and engendered a ‘lost sense of self’ (*n* = 11 studies). Ross and Green interpreted this as having a fragmented internal world—with some participants desiring to erase the real self and others wanting to erase the anorectic self (101). Still others could not distinguish between the two and needed help from therapists to externalize the illness (96). This distinction between the two ‘selves’ (healthy and anorexic) lies in direct contrast to the finding of AN being intertwined with the self. In some studies, participants described a feeling of both disconnection from the anorectic self and being intertwined with it, experiencing AN as an omnipotent voice that asserted its power over and gradually merged with the real self (91, 98).

### Anorexia nervosa as a tool

The third over-arching construct identified was the experience or use of restrictive eating behavior as a tool, usually to cope with the intense emotions and fears described previously. The AN provided a sense of self-anesthetization; of comfort and safety; and attenuated from the chaotic reality of an uncertain life or future. Four sub-themes were identified: avoidance/numbing, guardian/protector, control, and self-punishment/becoming needless.

The primary perceived reason for engaging with these behaviors was to avoid or numb, which was reported in half of all studies (*n* = 26). There were two ways in which participants used AN as a way to avoid or numb. Primarily, eating disordered behaviors distracted from and diffused feelings of pain, anger, fear and loneliness (77, 102–104). Many participants described being wholly absorbed by thoughts of food, calories and body weight such that any other thoughts or feelings were pushed aside. Secondly, being ill was itself used as a reason to avoid life and its attendant responsibilities, as well as to escape from one’s own unreasonably high expectations, where extreme perfectionism and ambition meant individuals felt pressure to perform in every area of their lives. Participants spoke of using the illness to elude this pressure and other performance anxieties; to miss school or events that were feared. The illness itself could provide a sense of purpose, something to focus wholly on. One participant acknowledged that “[living in institutions] reduces dramatically the amount of uncertainty that I’m faced with, and the chaos]” [(88), pp. 16–17].

Similarly, the illness was seen to be a guardian or protector (*n* = 24 studies); frequently personified as a companion who kept one safe and promoted a feeling of containment. More than 88% of participants in studies using thematic letter analysis mentioned it (38, 85, 93) and in Serpell’s study this was particularly salient for the adolescent participants (38). Participants frequently recounted being fearful children, wracked with anticipation of something bad happening, and AN was used to institute protective boundaries, to inure an individual to the “cruelty of the world” [(105), p. 171]. In some studies, participants spoke candidly of the inpatient environment reinforcing the protective power of the AN: “it’s safe, it’s comfortable really, cos you’re being looked after and there’s like, there’s no stresses of the outside world, which I think is an issue for me and I just, [hospital has] become normality, which is wrong really” [(91), p. 319].

The need for control is well-established in AN and is associated with high degrees of intolerance of uncertainty (30, 88, 106). Twenty-three studies addressed this sub-theme, with restrictive eating behavior described as providing consistency in life, giving structure to an individual’s day, creating an “own world with rules… [so that] the real world fades, matters less” (77). This was especially important in circumstances or times of great change (e.g., puberty, moving house or region, relationship breakdown). AN was seen to be predictable and to offer something tangible to measure oneself against: whether that be a number (weight) or a physical barometer of ‘hardness’. Participants spoke viscerally of this hardness: “I really need some fixed points in my life. I need to feel my skeleton…. When I don’t have access (to bones and skeleton), when there is something between what I feel when I touch myself and my inside, then I get scared. Things are blurred. … I want to be hard.” [(105), p. 169]. “In an otherwise limitless world, I was sure of my limit” [(105), p. 171]. Personal views on ability to control the illness varied according to illness discourse: those who felt greater personal agency in their weight loss tended to reject medical discourse pathologizing their behavior and more readily refuse treatment.

Finally, was the idea of becoming needless (*n* = 3 studies), underscored by a drive for self-punishment. Self-flagellation, whether that be depriving oneself of basic needs—not only food but in some cases, not allowing oneself to have showers or spend money, to rest, be “warm, [comfortable], allowed to sit on a soft chair” [(107), p. 7] or enjoy any pleasure. This detracted from the pain of AN and was interpreted by some researchers as a defensive system in which individuals turn inwards against the pain of having needs that are or were not met. This was frequently linked to experiences of rejection in childhood and ensuing trust issues. Denying all need for relatedness, juxtaposed against an excessive devotion to the needs of others, was first described as characteristic of AN by Bruch (108), and this was supported in the current literature. Participants appeared to be disgusted by their own ‘neediness’ and desired total psychic independence. Pleasure and joy were prohibited and this extended into all areas of basic human need, an extreme denial described by Robinson et al. as an almost ‘clinical frugality’ that worsened the longer someone was ill (109). Blackburn et al. wondered whether this extreme needlessness was a key phenotype of longstanding AN and may interact with or be a consequence of the effects of prolonged starvation (107).

### Internal conflict relating to anorexia

Life with AN was replete with conflict, which was the next construct identified. Participants reported rapid mood swings as well as conflicting feelings of pride and shame; consummate control and lack of control; feeling trapped by and trapping themselves within their illness by refusing to give it up.

Participants in 12 studies described feeling simultaneously in and out of control. This coincided with varying interpretations of illness discourse – that is, whether the illness was a functional tool and choice, or whether it was pathological. Acceptance of the medical discourse appeared to be positively correlated with experiences of emotional distress relating to eating disorder behaviors and increased motivation to change. Further, feeling ‘in control’ was inversely related to illness duration. Many participants depicted the journey of their AN much the same as would be described of addiction—starting as “a need, a compulsion to lose weight or gain control, then the habits you develop to fulfill that [compulsion] become ingrained and the compulsion is no longer owned by you […] Trying to ignore the compulsions you feel is like trying to get a heroin addict to turn down heroin- near on impossible.” [(102), p. 4]. “The hate sometimes is excruciating like a pain you want to leave, only this desire and control slots itself into every corner of my mind and I have allowed it to run parallel with my everyday life. [This is a] cell I have built for myself!”[(77), p. 654].

Participants in nine studies spoke of extreme functional impairment caused by their AN. This included: missed university lectures, inability to work, extreme fatigue and exhaustion, cognitive deficits, bankruptcy, homelessness, and inability to socialize. Functional impairment was inversely related to illness duration, and was frequently a cause of significant internal conflict, whereby individuals could see the devastating impact the illness was having on their life yet still wanted to hold on to it; or felt they were unable to let it go. In Robinson’s study of individuals with severe and enduring AN of more than 20 years duration, almost all were on state benefits (109). Most felt they hadn’t achieved what they would have been capable of had they not been ill. An earlier study by Arkell and Robinson used a mixed-methods approach to assess quality of life in individuals with Severe and Enduring AN showing similar scores between SE-AN and severely depressed primary care patients in subjective appraisal of quality of life, and impairment in living skills equal to patients with schizophrenia (77). It was the qualitative data in this study that explained why patients with SE-AN may score as poorly as severely depressed or psychotic patients on quality of life (QoL) and sociability scores, when they frequently demonstrate excellent communication skills and appear well-groomed and articulate. Here, interpersonal avoidance was identified as the driver of their isolation rather than any lack of skill; self-neglect a manifestation of self-denial (through punishing food and exercise regimes, extreme frugality etc.) rather than lack of personal hygiene or self-care skills (77). One impact of AN found to be most disturbing was the enormous cognitive load of the illness; the extent to which it occupied participants’ thoughts. They told of being wholly consumed by thoughts of food and dietary schedules. This myopic rigidity was obviously tedious to them, and meant they felt a lack of depth or richness in other areas of their lives, finding it difficult to pursue interests or hobbies. Participants described a life without meaning, ‘wooden’ and passionless, with no joy, ups or downs. “Activities become mindless rituals with no value or worth” [(77), p. 654]. Stockford et al. (46) outlined the circular nature of functional impairment in AN, whereby individuals are unable to work or study because of their illness, which then has a negative effect on self-efficacy and identity, exacerbating symptoms and impairing one further. The authors linked this self-perpetuating lack of self-efficacy with a greater need to hold on to the eating disorder identity (97).

There was significant cognitive dissonance when it came to feelings about recovery. Participants in nine studies spoke of the tension between wanting to recover and wishing to maintain aspects of the illness seen to be valuable. Ambivalence appeared to persist throughout the AN experience, with some participants believing they could recover from the AN but still remain an abnormally low weight. Others desperately wanted to recover but felt it was impossible, particularly if chronic. Surgenor (100) described an ‘idealized’ and ‘impassioned commitment’ to AN, where even those with longstanding histories of AN saw it as a source of immense enjoyment, a euphoria, an ascetic commitment to emaciation despite those same individuals speaking of the horrors of their illness. Charlotte says both she is “obsessing with food all the time… it’s terrible… it’s horrible,” and later, “you get stronger and stronger if you don’t eat… you can have a higher opinion of yourself, I guess. Being thin […] is the one enjoyment of life.” Emily is ‘repulsed’ by what she sees herself doing and would “rather be HIV-positive than have this life,” but at the same time says AN “makes you feel good” and remains committed to a low weight asserting that it is ‘healthy.’ Anne declares, “It’s really messed me up…I don’t want to do that anymore,” and then “everyone wants to continue to lose weight—you’re ok as long as the numbers are going down” [(100), p. 28].

### Interpersonal communication

Themes relating to interpersonal communication were significant and constituted the penultimate construct identified in the review.

The first sub-theme relating to interpersonal communication was difficulty verbalizing distress (*n* = 10 studies). AN has been described as an internalizing illness (110–112), and this was supported by the review, with participants reporting significant difficulties expressing negative emotion (predominantly anger and sadness). AN was seen as way to communicate these difficulties, as were overt self-harm behaviors, risk taking and the act of destroying belongings. Some participants who experienced overwhelming emotion spoke of growing up in families with low expressed emotion and/or emotion denial. Others had witnessed or been on the receiving end of extreme anger as children or had parents or caregivers who found it very difficult to manage their own emotions. While participants reported feeling distressed ‘most of the time’, they were either afraid of being a burden on others and thus inhibited expression of this by ‘masking’ (putting on a happy face) or were unable to find the language to express this emotion. Some authors questioned whether individuals with AN are truly alexithymic or whether they simply need prompting to find the courage to discuss their emotions (78).

Participants in eight studies described difficulties with interpersonal relationships, rooted largely in an inability to interpret the intentions of others, and a tendency to over-analyze emotional reactions and non-verbal cues (104, 113). This heightened sensitivity appears to contribute to a profound sense of uncertainty around social interaction (88). Their rigid and inflexible behaviors aggravate the problem, leading many to deliberately avoid social interaction, in a manner that could be considered clinically socially phobic (104). While these participants appeared to have developed protective interpersonal strategies to guard from emotional pain by keeping people at a safe distance (114), for others there did not appear to be any social skill deficit and instead the isolation seemed to be a result of the progression of the illness. “I’m really surprised actually, when people talk about their friends and all that—because I have no friends! For the past 10–12 years, I haven’t done anything, I haven’t gone out with anyone, I haven’t spoken to anybody, in a social context, so like [patients who’ve had] families, that absolutely astounds me!” [(104), p. 851]. Fox and Diab observed that the experience of living in the hospital may exacerbate participants’ interpersonal difficulties, as it meant spending large periods of time away from family and peers (103). Kyriacou observed stark differences between the empathy experience of participants and the perception of those around them: individuals felt they were overly sensitive to the emotions of others, whilst parents and clinicians described them as highly intelligent but showing substantive socioemotional lack: frequently preoccupied with the responses of others and seeming to attribute self-responsibility to any negative interaction or paraverbal cue (104). The same parents and clinicians described a complete inability of the patient to comprehend others’ emotional state in relation to the seriousness of their illness and the concern it caused (104). Equally, O’Shaugnessey et al. described the difficulties those with AN had in imagining the perspective of others (114). This conflicted with reports of individuals in other studies, who were deeply burdened by the pain their illness caused loved ones.

Participants in just six studies expressed a need for social approval, and this typically stemmed from low levels of self-esteem and self-efficacy. Sense of self-worth was gained from what other people thought, and individuals tended to engage in mind-reading, convinced others thought they were ‘disgusting,’ ‘stupid’ or a ‘failure.’ Any feeling of being judged by others manifest in fears about body image and could change in an instant the way an individual experienced their body image. Any real or perceived negative evaluation could make an individual ‘grow’ spontaneously, as if their bones disappeared before someone’s eyes; they could put their hand on their hip and feel that their hipbone had disappeared (115).

### Corporeality

The final overarching concept identified was corporeality. This concept encapsulated sub-themes involving both tangible and metaphorical experiences. The two dominant sub-themes here were a feeling of bodily disconnection and a need to disappear.

Participants in seven studies described a ‘split’ between mind and body, which were experienced as two distinct entities, the latter ultimately uncontrollable by the former as was typically desired. Those expressing this sentiment appeared to be surprised that their body existed to touch at all, feeling that it bore no relation to their cognitive reality: “I remember sort of…looking in the mirror and actually being surprised that I saw a form in the mirror and not just nothingness” [(116), p. 145]. In Bates’ (117) study of metaphors associated with AN, the body was commonly described as an ‘object’ or ‘cage’ that was problematized—seen as a barrier to participants’ true, inner self. Such dissonance between body and mind (or the body and the “true self”) caused great distress. Strong associated feelings of disgust meant the body was something to be fought against. There was an awareness of the link between food and connection to being present, to people, and to life, and hence something to be avoided. Embracing the anorexia allowed one to “not be here” [(89), p. 231], which was seen as desirable to participants struggling with an existential splitting (99).

Linked to this was a need to disappear (*n* = 7), which served two paradoxical aims: primarily to ameliorate the ‘too muchness’ associated with strong feelings of anxiety (118) and contrarily, as a sort of disappearing act that positioned the individual as an otherworldly-being ‘disappearing to be seen,’ embracing an ethereal rhetoric of life on the edge. This was interpreted by study authors as an enacted postmodern erasure of the submissive, ‘impossibly contained’ and ‘inferior’ female (116); a conscious disembodiment, where AN acts as a “‘selfing device”: an apparatus of becoming and an arena of being a heroic self.’ [(119), p. 290]

The third sub-theme identified was hunger as a physical experience both painful and pleasurable (*n* = 3 studies). Hunger gave participants a sensation similar to a drug ‘high,’ where an individual feels as though they are standing ‘outside’ their body and thus transcending reality (119). One participant described the highs she experienced when her blood sugar level dropped after binging and purging, and when she hadn’t eaten enough as a dizzy, light-headedness (80).

The last sub-theme identified was the need to be empty (*n* = 3 studies), which was usually associated with feelings of being ‘too much.’ ‘Too much’ could refer to the physical space an individual occupied, or to feelings of overflowing emotions that were difficult to contain (105). Whilst participants in few studies reported on feelings of fatness, those who did described internal bodily sensations (feeling “hot,” feeling “heavy” and “crawling out of your skin”) closely linked with heightened emotion [(120), p. 369, (121)]. In most studies, feeling ‘fat’ was a blanket description for something else, captured elsewhere in the synthesis.

## Discussion

Anorexia Nervosa is an internalizing illness frequently suffered by achievement-oriented individuals with anxious or obsessional traits who report high levels of fear in childhood and turn inwards for a solution to a myriad of intrapsychic pain. What is notable about this reading is the effectiveness of restrictive eating behaviors in anesthetizing against it (particularly fear and anxiety) and ostensibly the depth to which the illness provides an individual with a strong sense of self, notwithstanding the extraordinary pain, profound solitude and significant impairment it causes. The metasynthesis endorses certain enduring clinical assumptions of the illness whilst challenging others. Dominant and reciprocal sub-themes included fear and anxiety, AN as role or intertwined with identity, AN as avoidance mechanism and AN as guardian/protector. Other themes frequently associated with AN such as self-punishment, need for social approval and feelings of fatness were less common.

AN has long been associated with emotion dysregulation and harm avoidance (48, 128–131), however, has largely been measured quantitatively using researcher-derived constructs. The strength of this association and its clear phenomenological implication is clearly supported here, with a majority of participants suggesting their AN was both caused by and exacerbated an inability to manage or understand intense emotion. Overwhelmingly, this emotion was fear and anxiety, which was linked to almost every other dominant sub-theme, including the need to avoid or numb that fear, the view of AN as a guardian or protector, the desire to achieve greatly to appease the need to be perfect, and the drive for ultimate control through limiting oneself and one’s boundaries. There were also strong feelings of sadness, though this was usually in response to the effects of the illness itself. Individuals are typically highly independent and appear to want to ‘solve’ their emotions on their own, masking negative affect in front of others due to an inability to verbalize distress, fear of anger or rejection if displaying emotion, trait sensitivity, and worries of placing undue burden on others.

There is growing evidence of a link between traumatic experiences and severity of eating disorder presentation (107, 132), and a number of participants in this study reported experiences of rejection, neglect, abandonment or bullying as children, which conflated with temperamental trait-sensitivity to withdraw individuals from their own needs and from relationships with others. Participants appeared to want to become needless, frugally rejecting basic human needs including pleasure and warmth. They wanted to ‘become hard,’ have a firm outline that protected, or ‘buffered’ against the cruelty of the world and attack from others (104, 105). AN gives them this. Unable to trust others or be vulnerable, it provides some sense of internal safety and solution to loneliness: a means of self-soothing “but at the cost of a desolate feeling of being trapped within, unable to reach out or be reached” [(98) p. 11, (122)]. The paradox between a strong need to be looked after and highly developed interpersonal strategies to keep people and relationships at a distance typifies the push–pull nature of the illness (93, 114). Individuals long for closeness but are fearful or dismissive of it. This phenotype was clearly demonstrated here and is reported in studies linking AN with disorganized or unresolved/dismissive attachment (133–136).

From the reciprocal translation there emerged a cyclical picture of the AN experience in which difficulties regulating or knowing what to do with confusing emotion is root. In an attempt to manage or diffuse the distress, individuals starve themselves (shown to regulate negative affect) (79, 137, 138) or engage in other self-destructive behaviors (99). Using eating behaviors to avoid emotions creates a vicious cycle in which one becomes increasingly out of touch with their emotions, and as the illness progresses, so too does emotional dysfunction (113). This supports Schmidt and Treasure’s cognitive-interpersonal maintenance model of AN, which stipulates that avoidance of emotion becomes synonymous with eating disordered behaviors, creating a vicious cycle where an individual becomes increasingly detached from their emotions (62).

Appearance was alluded to primarily as a reflection of the ‘success’ of one’s eating disordered behaviors: when individuals felt threatened by their emotions, others, or the failure to meet their own expectations, their unconscious reaction was to evaluate their appearance negatively. When they felt they had not ‘obeyed’ their anorexic rules, they evaluated their appearance negatively. Further, certain behaviors tended to link with specific emotions: a number of studies observed a link between binge/purge behavior and anger and restrictive behavior and fear (77, 78, 122). The lexicon around types of emotion and their influence on differential behavior in this study demonstrates clear benefits of qualitative, open-ended research. Instead of body-centric language related to feelings of bodily disgust and shame or feelings of fatness, what was heard in these studies was emotion-centric language. This supports research suggesting individuals with AN tend to mislabel averse physical and emotional states as ‘being fat’ (105, 121, 139, 140), meaning researcher-derived assessment tools which measure only weight, shape and body-related constructs may fail to capture what AN really is and mislabel it in the same way sufferers do themselves.

Researchers have explored AN under an anxiety disorder framework, noting the presence of intense fear and the function of safety behaviors, as well as psychotic features (141, 142). Certainly, frequent comorbidity between eating and anxiety disorders has been established (13, 33, 106), however, the extent to which this review speaks of the ripple effect of underlying anxiety on almost every part of the anorectic experience; the use of anorectic behaviors constituting a safety behavior; and the apparent delusionality of some participants who spoke of ‘growing or expanding’ spontaneously provides some support for the conceptualization of AN as a type or idiosyncratic expression of anxiety disorder (122, 141).

The synthesis can be read from an Eriksonian perspective of identity formation. The body, a vessel upon which confusing emotion is displaced (87, 122) is concretized in the process of ‘becoming’ or developing an identity. AN frequently onsets in adolescence, when individuals undergo separation/individuation from their carers and a transformation of self (role confusion) frequently frightening and constituting a loss (143). This separation conflates with fear of uncertainty or low self-esteem resulting in an attempt to control the vessel of confusing emotion (body). Further, the body is objectified in Western culture as a canvas to perfect and is thus the perfect conduit in which to become ‘other’; to control or project an image or self, and through this, a concrete identity (144). Under this lens, an individual relinquishing their AN would re-enter the role confusion stage of identity formation and re-live all of its extant anxieties. Given individuals with AN have been frequently found to be intolerant of uncertainty and low in self-esteem (though the latter was not a major theme here), it is unsurprising they may avoid relinquishing a strong sense of identity (30, 93), and we can see the act of being ‘refed’ or gaining weight may be a powerful existential threat, compromising their very sense of self and only safe way to ‘be’ in the world (91, 103, 145).

Several studies conceived of the AN identity functioning initially to improve self-esteem through attaining bodily perfection and control over biological need. This identity provides, at least at first, a sense of empowerment. In this way, the act of ‘becoming’ ill can be seen as somewhat deliberate and breeds the egosyntonic tousle famous of AN (28, 146). Here, the blurring of the self and mental illness is compounded by the seemingly purposive nature of eating disorder behaviors and the sense of empowerment, belonging or community one feels with fellow ‘sufferers’ (89). However, the more the sense of self is entwined with AN, the more chronic a patient becomes (127, 145). Prolonged starvation leads to feelings of hopelessness and despair, and exacerbation of the intense emotions (particularly shame, sadness, loneliness) it originally intended to ‘fix.’ As individuals become more functionally impaired, particularly if they have frequent and intense contact with health services, their AN illness identity solidifies, filling an ever-widening gap in their sense of self (51, 91). Williams et al. questioned whether healthcare professionals should be treating AN as an illness or an identity, arguing that current biomedical discourse may not reflect the lived experience of the condition (96), while others appear to recommend an illness-identity-limiting person-centered approach outside of insulated settings (51, 91, 96, 147).

This review is suffused with references to pathological perfectionism: the extreme pursuit—sometimes to the detriment of all other personal values—to pursue those that are morally revered such as discipline and self-control. This is a core feature of some of the earliest recorded cases of AN (148, 149). Authors emphasized the difficulty of disentangling the cognitive effects of starvation (rigidity) from premorbid personality traits, however, the diminishing effect of need for achievement and perfectionism as the illness progressed was clear, with those individuals in the early stages of the illness appearing to have the strongest need for excellence and be more likely to reject illness discourse (85, 127).

Further, it appears that enduring anorectic behaviors may over time become only loosely associated with their original intent, solidified instead by gross habit. Whilst AN was frequently about gaining ‘control,’ there was a clear inability to control compulsion to engage in anorectic behaviors in much of the qualitative data, particularly as the illness progressed. Auto-addiction and activity-based anorexia models propose that abnormal activation of the endogenous opioid system in response to starvation and over-exercise in individuals with AN makes repeated engagement in the behavior similar to chronic administration of opiate drugs (150–152). Certainly the described ritualistic and compulsive behaviors in this review combined with an apparent inability to cease behaviors despite adverse consequences suggest the experience of anorexia in many ways resembles the experience of typical addiction (153, 154).

Participants in the studies were at varying stages of their illness journey. A large *N* and reciprocal translation allowed us to discern similarities and differences in experience across the stages. Individuals in the latter stages of the illness in many cases had done the therapeutic work to address the etiological factors of their psychopathology and were still unable to feed themselves ‘normally.’ The enduring strength of habit, the disruption the illness causes to their lives, and the existential break in their sense of self, all appear to prolong the illness long after the root of the anorectic behaviors has been resolved, entrenching illness identity (97). Compounded by ongoing dislocation from peers and ‘normal’ psychosocial development, individuals who develop an enduring course may do so as a result of the residual effects of the AN as much as of the psychopathology itself (155).

In sum, the metasynthesis finds the Anorexia Nervosa experience to be rooted in profound fear and emotion dysregulation or alexithymia; typified by “obsessional character structure, interpersonal insecurity, minimization of affect, excessive conformance and regimentation of behavior, and heightened industriousness and responsibility” (129). A solution to concomitant anxieties, AN is a remarkably effective avoidance mechanism; a prized but somewhat lonely quest for extraordinary achievement. It’s extremely powerful anaesthetizing effect inures to resultant feelings of sadness and significant adverse physical and psychosocial consequences, but ultimately traps an individual in a joyless and regimented life they feel safe in but despise. They may see themselves within the disease at once both heroic and pitiable, but ultimately are looking for a way to exist within their emotions; to create an identity syntonic with a self that has been lost. Thus, they survive and are managed through and within Anorexia, which permeates every waking thought, decision and interpersonal interaction; a ‘profound biological solution to existential problems’ (156).

## Conclusion

The review identified six key constructs of the Anorexia Nervosa experience: “Emotion as overwhelming,” “Anorexia as a tool,” “Identity,” “Internal conflict relating to Anorexia,” “Interpersonal Communication,” and “Corporeality.” Each had their own, sometimes contradictory, themes exploring an individual’s cognitions, conceptualization and emotional processes of the illness which, when translated reciprocally and refutationally, provide a detailed phenomenological picture of the ‘neurotic’ condition Anorexia Nervosa.

In AN treatment, there is no doubt nutritional and medical intervention saves lives in cases of severe medical instability and low BMI. However, the extent to which the illness is perceived to protect an individual from a chaotic and threatening world and the degree to which avoidance and illness identity exacerbate it, may be reinforced by weight/shape-focused therapeutic models and repeated engagement with insulated treatment settings which provide a rigid framework upon which to build a self (146). Separation of the self from the AN is imperative for recovery (91, 125), necessitating the development of emotion identification, healthy emotion expression and social skills, and opportunities to develop multiple discursive constructions of the self (e.g., as sister, friend, athlete, worker, student, traveler) (117). There should be a careful balance between estimated imminent medical risk and over-hospitalization, an imperative to be caregiving whilst fostering independence and a purpose outside of the illness.

The importance of the therapeutic relationship in increasing motivation, re-writing internal narratives and building self-efficacy and self-compassion for the experience of intense emotion is clear and underscores the potential benefits of a broader range of underutilized therapies such as compassion-focused and exposure therapies, interpersonal and psychodynamic therapies (85, 99, 104, 113, 127, 139, 157–159). While Cognitive Behavior Therapy demonstrates its efficacy in challenging unhelpful thoughts and modifying shape and weight concerns (160, 161), it may be that the reality of the strength of this illness, the intensity of the associated fear response and the habitual avoidance at its base necessitates a more dynamic treatment approach, ideally delivered within the person’s life (i.e., on an outpatient basis).

Limitations of the metasynthesis must be addressed. Participants in the studies were predominantly Caucasian and female. Individuals of other genders and/or ethnicities may experience AN differently. Further, the heterogenous nature of recruitment across studies means participants were at different stages of their illness, and whilst this offers a picture of the illness journey, it may have skewed results in favor of earlier or later stage illness. Many of the sub-themes are conceptually similar, and all in a constant state of flux or transience. The derivation of third-order constructs by nature requires the researcher to use subjective judgment to discern appropriate constructs and sub-themes from conceptually similar data. The combined lived, clinical and research experience of the authors and collaborative discussion around the appropriateness of the adopted constructs aimed to mitigate this inherent subjectivity and arrive at consensus. Further, the frequency of mentioned sub-themes and third order constructs may have been skewed toward studies examining certain themes, as there was theme heterogeneity across studies (however, given only ten papers looked specifically at emotions and this was the most dominant sub-theme, this is unlikely to have impacted results).

Lastly, the volume of studies and broad research question means there were complex interpretive possibilities. This is to be read as an interpretation of the named authors only and understood in the context of their reflexivity statement. Further phenomenological metasynthesis should be undertaken in other eating disorder diagnostic groups as distinct from AN to better understand transdiagnostic or heterogenous illness experiences for more effective therapeutic targets. Further, collaborative qualitative research with consumers should be more frequently employed as a means to dissect all aspects of eating disorder psychopathology. Only from multiple lenses and with deep philosophical enquiry may we begin to understand the complex experience of Anorexia Nervosa.

## Data availability statement

The original contributions presented in the study are included in the article/Supplementary material, further inquiries can be directed to the corresponding author/s.

## Author contributions

EB: conceptualization, methodology, formal analysis, investigation, writing – original draft, visualization, and project administration. PA: methodology, investigation, formal analysis, writing – review and editing. AH: investigation and writing – review and editing. ST: conceptualization and writing – review and editing. SM: conceptualization, writing – review and editing, and supervision. All authors contributed to the article and approved the submitted version.
