# Supplementary material for: ‘In an otherwise limitless world, I was sure of my limit.’ Experiencing Anorexia Nervosa: A phenomenological metasynthesis
Source: Front Psychiatry. 2022 Aug 1;13:894178. doi: 10.3389/fpsyt.2022.894178 (PMC9376373; doi:10.3389/fpsyt.2022.894178)
Supplement: Supplementary file 1 [file Table_1.DOCX]

**APPENDICES**

**Appendix A.** Search syntax used in the systematic search of electronic databases

| *Terms relating to Anorexia Nervosa*  Eating disorders  Anorexia Nervosa | Eating disorder*  Anorexi*; Atypical Anorexi* |
| --- | --- |
| *Terms relating to phenomenology*  Phenomena  Experience  Cognitions  Emotions  Being  Living with  Feeling | Phenomen*  Experience*  Cognition*  Emotion*  Being  Lived; Living  Feeling* |
| *Terms relating to qualitative research*  Qualitative research  Grounded theory  Ethnography  Ethnomethodology  Hermeneutic  Content analysis  Focus Group  Thematic analysis  Discourse analysis | Qualitative and qualitative stud*  Grounded theor* and grounded stud*  Ethnograph*  Ethnomethodolog*  Hermeneutic  Content analysis  Focus group*  Thematic* and theme*  Discourse analysis |

**Appendix B.** Studies included in the meta-synthesis

| **Author, Year, Country** | **Title** | **Aim** | **Recruitment setting** | **Participant demographic characteristics; sample size (*n*)** | **Qualitative methods** |
| --- | --- | --- | --- | --- | --- |
| **1. Arkell and Robinson, 2008, UK (Arkell & Robinson, 2008)** | A pilot case-series using quantitative and qualitative methods: biological, psychological and social outcome in severe and enduring eating disorder | To explore the level of disability and quality of life in participants with severe and enduring eating disorder (anorexia nervosa) | Specialist eating disorders unit, North London | Individuals with SE-AN; 10 females, 1 male, Mean age 37.7 (SD 8); 11 | Self-report survey and unstructured writing task; |
| **2. Bates, 2015, Worldwide (Bates, 2015)** | “I am a waste of breath, of space, of time”: Metaphors of self in a pro-anorexia group | To examine how individuals identifying as anorexic view and present the self | Online | Individuals self-reporting anorexia nervosa; 757 | Content analysis;  Metaphor identification procedure |
| **3. Blackburn et al., 2020, Ireland (Blackburn et al., 2020)** | Becoming needless: A psychoanalytically informed qualitative study exploring the interpersonal and intrapsychic experiences of longstanding anorexia nervosa | To explore the interpersonal and intrapsychic experiences of individuals living with longstanding anorexia and considered how such experiences might influence barriers to recovery | Online advertisement through national eating disorder charity | Females aged 22-44, formally diagnosed with AN; 6 | Psychoanalytically-informed;  Qualitative content analysis |
| **4. Broomfield, Rhodes & Touyz, 2021, Australia (Broomfield et al., 2021)** | How and why does the disease progress? A qualitative investigation of the transition into long-standing AN | To track processes that occur as the AN illness progresses | Social media and clinician networks | Females over aged 18 with current or former DSM-5 AN diagnosis; 11 | Narrative inquiry; Photovoice method & interview |
| **5. Button & Warren, 2001, UK (Button & Warren, 2001)** | Living with anorexia nervosa: the experience of a cohort of sufferers of anorexia nervosa 7.5 years after initial presentation to a specialised eating disorders service | To further understand how sufferers of AN view the disorder, how it affects their lives and how they experience treatment and help. | Specialist ED service | Individuals diagnosed with AN 7 years prior, mean age 27.9; 36 | Semi-structured interview; |
| **6. Chan & Ma, 2003, HK (Chan & Ma, 2003)** | Anorexic body: a qualitative study | To explore the anorexia nervosa (AN) patient's subjective experience in family therapy by employing a qualitative inquiry | Home of individual | Female individual aged 25, AN patient; 1 | Video & writing task; |
| **7. Conti, 2016, Australia (Conti, 2016)** | “I don’t think anorexia is the way out”: Reconstruction of meaning in women’s narratives of anorexia nervosa over 10 years | To explore how nine women responded over 10 years to the realities of their lived experience, how they ascribe meaning to their experiences and negotiate their identities within the illness. | Advertisement on news story | Females aged 21- 44 self-identified AN; 9 | Critical discursive analysis |
| **8. Cooper et al., 2007, UK (Cooper et al., 2007)** | The experience of ‘feeling fat’ in women with anorexia nervosa, dieting and non-dieting women: an exploratory study | To provide a preliminary, systematic exploration of some features associated with the experience of ‘feeling fat.’ | Community advertising & clinician networks | Females with AN, Mean age 29.6  (dieting females and non-dieting females were excluded from our analysis); 16 | Semi-structured interview;  Coding/exploratory study |
| **9. Cruzat-Mandich, 2017, Chilé** (Cruzat‐mandich et al., 2017) | From eating identity to authentic selfhood: identity transformation in eating disorder sufferers following psychotherapy | To explore the development of the identity configuration process of Chilean female patients with an eating disorder (ED) following successful psychotherapeutic treatment | Eating disorder service | Females in treatment for AN or BN; 20 | Semi-structured interview; Grounded theory approach |
| **10. Dahlborg Lyckhage et al., 2015, Sweden (Dahlborg Lyckhage et al., 2015)** | Young women with anorexia nervosa: writing oneself back into life | To describe how young women living with self-identified anorexia narrate about their lives by blogging. | Internet | Female bloggers self-identifying as having AN; 13 | Internet blog analysis; Qualitative Content Analysis |
| **11. Espeset et al., 2012, Norway (Espeset et al., 2012a)** | Fluctuations of Body Images in Anorexia Nervosa: Patients' Perception of Contextual Triggers | To explore which everyday contexts that patients with AN themselves associate with fluctuations in body image | Four clinical institutions specialising in ED or general psychiatry | Women aged 20-35 diagnosed with AN; 32 | Semi-structured interview; Grounded theory methodology |
| **12. Espeset et al., 2012, Norway (Espeset et al., 2012b)** | The link between negative emotions and eating disorder behaviour in patients with anorexia nervosa | To explore how patients with AN manage the aversive emotions sadness, anger, fear and disgust, and how they link these experiences to their eating disorder behaviours. | Specialist eating disorders service | Individuals diagnosed with AN, aged 20-39 (M 29.1); 14 | Semi-structured interview; Grounded theory methodology |
| **13. Faija et al., 2017, UK (Faija et al., 2017)** | The role of pride in women with anorexia nervosa: a grounded theory study | Explore experiences of and reflections of pride in women with a current or past diagnosis of AN. | Specialist eating disorders unit; and a self-help organisation | Females 18-61 (M 29.67) diagnosed with AN; 21 | Semi-structured interview; Constructivist Grounded Theory |
| **14. Freedman et al., 2006, Canada (Freedman et al., 2006)** | Understanding anorexia nervosa through analysis of thematic content of letters in an adolescent sample | Aims to understand how adolescents relate to their illness | Inpatients and outpatients at BC Children’s Hospital | Mean age 16.7; 27 | Letter writing task; Grounded theory methodology |
| **15. Fox, 2009, UK (J. R. E. Fox, 2009)** | A qualitative exploration of the perception of emotions in anorexia nervosa: a basic emotion and developmental perspective | To provide a rich and thorough account of emotions within anorexia nervosa. | Regional eating disorders service in UK – inpatients and outpatients | Females aged 18-51; 11 | Semi-structured interview; Grounded theory methodology |
| **16. Fox & Diab, 2015, UK (J. R. Fox & Diab, 2015)** | An exploration of the perceptions and experiences of living with chronic anorexia nervosa while an inpatient on an eating disorders unit: an interpretative phenomenological analysis (IPA) study | To explore the perceptions and experiences of individuals living with chronic AN. | Specialist eating disorders unit (inpatient) | Individuals diagnosed with AN, aged 19-50 (M27); 6 | Semi-structured interview;  Interpretive Phenomenological Analysis |
| **17. Fox, Larkin & Leung, 2011, UK (A. P. Fox et al., 2011)** | The personal meaning of eating disorder symptoms: an interpretive phenomenological analysis | To explore the personal meaning of eating difficulties | BEAT (UK charity) advertisements | Females aged 18-30; 8 | Semi-structured interview; Interpretive Phenomenological Analysis |
| **18. Godier and Park, 2015, UK (Godier & Park, 2015)** | Does compulsive behaviour in anorexia nervosa resemble an addiction? | To investigate to parallels between AN and addiction in terms of compulsive behaviour/compulsivity | Email, internet and poster advertisement (incl BEAT UK) | 39 female, 1 male; self-reported AN, mean age 28; 40 | Self-report survey and interview; Thematic Analysis |
| **19. Gooldin, 2008, Israel (Gooldin, 2008)** | Being anorexic: hunger, subjectivity, and embodied morality | To provide an anthropological exploration of anorexia from within; that is, as a situated embodied knowledge of anorexic women anchored in concrete lived experiences. | Outpatient eating disorders unit at children’s hospital | Females; 7 | Ethnography |
| **20. Granek, 2007, Canada (Granek, 2007)** | ‘You’re a whole lot of person’ – Understanding the Journey through Anorexia to Recovery: A Qualitative Study | To examine the subjective experience of anorexia nervosa | Community word-of mouth and flyer advertising | Females who had experienced AN for at least 1 year, aged 25-30 years old; 5 | Open-ended interviews; Grounded theory methodology |
| **21. Hannon et al., 2017, UK (Hannon et al., 2017)** | The patient experience of illness, treatment, and change, during intensive community treatment for severe anorexia nervosa | To explore experiences of intensive community treatment, illness and change among patients with severe anorexia nervosa (sAN), particularly seeking to understand the processes involved in change and inability to change. | Individuals in intensive outpatient treatment | Females with severe AN aged 23-39; 5 | Semi-structured interview; Interpretive Phenomenological Analysis |
| **22. Higbed & Fox, 2010, UK (Higbed & Fox, 2010)** | Illness perceptions in anorexia nervosa: a qualitative investigation | Explore illness perceptions of people actually suffering with AN, as this can impact coping and treatment outcomes | Inpatients and outpatients at specialist ED unit north of England | Individuals diagnosed with AN aged 16-53 (M26); 13 | Semi-structured interview; Constructivist Grounded Theory |
| **23. Hope et al., 2011, UK (Hope et al., 2011)** | Anorexia nervosa and the language of authenticity | To investigate patients experiences of and attitudes towards AN and to explore specific areas relevant to decision making and competence. | 4 specialist ED services across England | ? Aged 15-26; 29 | Semi-structured interview; Grounded Theory Analysis |
| **24. Konstantellou et al., 2019, UK (Konstantellou et al., 2019)** | The experience of intolerance of uncertainty for young people with a restrictive eating disorder: a pilot study | To advance our understanding of the relationship between IU and restrictive EDs by providing insight into young people’s subjective experiences of uncertainty. | Specialist eating disorder service | Young people aged 12-18; 13 | Focus groups; Interpretative Phenomenological Analysis |
| **25. Koruth et al., 2012, UK (Koruth et al., 2012)** | A grounded theory exploration of the onset of anorexia in adolescence | Explore individuals’ point of view of the early stages of their anorexia. | Inpatients and outpatients CAMHS | Young people ages 13-17, 7 females and 1 male; 8 | Semi-structured interview; Grounded Theory Methodology |
| **26. Kyriacou et al., 2009, UK (Kyriacou et al., 2009)** | Comparing view of patients, parents and clinicians on emotions in anorexia: a qualitative study | To explore the most salient issues pertaining to emotions and social cognitions in AN. | Patients diagnosed with AN; their clinicians and carers | Individuals diagnosed with AN (check), mean age 26.8; 6 | Focus groups; Thematic Analysis |
| **27. Lavis, 2018, UK (Lavis, 2018)** | Not eating or tasting other ways to live: a qualitative analysis of living through and desiring to maintain anorexia | To explore what anorexia does for and to an individual, from their own perspective. To ‘explore the experiences of eating disorders beyond the lens of emaciation.’ | Specialist eating disorders unit and online advertising. | Not reported; 60 | Iterative Thematic Analysis |
| **28. Leppanen et al., 2021, UK (Leppanen et al., 2021)** | Critical incidents in Anorexia Nervosa: perspectives of those with a lived experience | To shed light on both positive and difficult emotional experiences in AN by retrospectively exploring critical life events identified by those with a lived experience of AN in the acute phase of the illness. | Flyer advertising on university campus | All genders aged 16 and over with current or past AN; 34 | Critical Incident Technique questionnaire |
| **29. Major et al., 2019, UK (Major et al., 2019)** | The experience of feeling fat for women with anorexia nervosa: an interpretive phenomenological analysis | To explore participants experience of ‘feeling fat’ and its implications for clinical treatment | NHS | Females aged 18-50; 7 | Semi-structured interview; Interpretive Phenomenological Analysis |
| **30. Malson, 1999, UK (Malson, 1999)** | Womæn under erasure: anorexic bodies in postmodern context | To explicate the discourses and discursive resources deployed in constructions of `anorexia',`femininity' and embodied subjectivity | NHS and self-nomination | Females, diagnosed and self-diagnosed with AN; 23 | Semi-structured interview; Discourse analysis |
| **31. Marzola et al., 2015, Italy (Marzola et al., 2015)** | A qualitative investigation into anorexia nervosa: the inner perspective | Explore the meaning patients attribute to their AN. | Day hospital | Females diagnosed with AN; 34 | Letter-writing task; Thematic Coding |
| **32. Mulkerrin et al., 2016, UK (Mulkerrin et al., 2016)** | How well does anorexia nervosa fit with personal values? An exploratory study | To explore personal values among individuals with AN, with a particular focus on the ‘fit’ between participants values and their AN. | Two London-based ED services | Females aged 20-40; 8 | Semi-structured interview; Interpretive Phenomenological Analysis |
| **33. Nordbo, 2006, Norway** (Nordbø et al., 2006) | The meaning of self-starvation: qualitative study of patients’ perceptions of anorexia nervosa | To systematically explore the meaning that patients with AN attribute to their anorectic behaviour. | Clinician networks | Females in treatment for anorexia nervosa, aged 20-34 (M 25.5); 18 | Phenomenological Analysis |
| **34. O’Connell, 2018, Australia** (J. E. O’Connell et al., 2018) | Delusion-like beliefs in anorexia nervosa: an interpretative phenomenological analysis | To qualitatively explore beliefs held by AN patients and the consequences of holding such beliefs. | Inpatients diagnosed with AN | Female aged 19-31; 5 | Semi-structured interview; Interpretive Phenomenological Analysis |
| **35. O’Connell, 2021, UK** (O’Connell, 2021) | Being and doing anorexia nervosa: An autoethnography of diagnostic identity and performance of illness | To narrate and analyse the author’s experience of and relationship with her anorexia nervosa and the constructions of it she encountered | Self | Female, recovered from AN; 1 | Autoethnography |
| **36. O’Shaughnessy et al., 2013, UK** (O’Shaughnessy et al., 2013) | A narrative study of the lives of women who experience anorexia nervosa | To explore the subjective experiences of women over the age of 20 diagnosed with anorexia. | Individuals receiving treatment at a local eating disorders service. | Females aged 21-35; 4 | Semi-structured interview; Narrative Analysis |
| **37. Petersson, Gullbing and Perseius, 2021, Sweden (Petersson et al., 2021)** | Just like fireworks in my brain – a Swedish interview study on experiences of emotions in female patients with eating disorders | To explore the experiences of emotions as narrated by women with eating disorders at an out-patient clinic in Sweden | Outpatient ED clinic in Southern Sweden | Females aged 18-51; 7 with AN (9 total participants) | Thematic analysis; Semi-structured interview |
| **38. Rance et al., 2016, UK (Rance et al., 2017)** | The anorexia nervosa experience: shame, solitude and salvation | To understand the lived experience of AN/its everyday phenomenology. | BEAT UK advertisement & NHS regional counselling services | Female aged 18-50; 12 | Semi-structured interview; Thematic Analysis |
| **39. Rich, 2006, UK (Rich, 2006)** | Anorexic dis(connection): managing anorexia as an illness and an identity | To examine how young women make sense of the various social constructions of EDs at an everyday level | Residential treatment centre | Female; 7 | Semi-structured interview; Ethnography |
| **40. Robinson, 2015, UK (Robinson et al., 2015)** | Severe and Enduring Anorexia Nervosa (SEED-AN): A qualitative study of patients with 20+ years of anorexia nervosa | To investigate how patients with long term eating disorders manage their clinical problems | Specialist eating disorders service | Individuals who had suffered from AN for 20-40 years; 8 | Semi-structured interview; Thematic analysis |
| **41. Ross & Green, 2010, UK (Ross & Green, 2011)** | Inside the experience of anorexia nervosa: a narrative thematic analysis | To understand the experience of two women with chronic anorexia, and how they experience inpatient treatment | Specialist eating disorder service; outpatients (former inpatients) | Females over 18; 2 | Thematic Analysis |
| **42. Serpell et al., 1999, UK (Serpell et al., 1999)** | Anorexia nervosa: friend or foe? | To examine anorexics’ attitudes towards AN. | Specialist eating disorder unit (unsure whether in/outpatient) | Median age 24.1; 18 | Grounded Theory Methodology/Coding |
| **43. Skårderud, 2007a, Norway** (Skårderud, 2007a) | Eating one’s words, part I: ‘concretised metaphors’ and reflective function in anorexia nervosa—an interview study | To understand the body’s symbolic role—embodiment in AN. | Outpatients in private practice | Female aged 16-35; 10 | Semi-structured interview; metaphor analysis |
| **44. Skårderud, 2007b, Norway** (Skårderud, 2007b) | Shame and pride in anorexia nervosa: a qualitative descriptive study | To define shame and describe types and subtypes of shame and their relations to symptoms and meaning in anorexia nervosa. The study will also describe the possible role of pride, as a contrasting emotional and cognitive experience. | Outpatients in private practice | Female aged 16-39; 13 | Semi-structured interview; coding analysis |
| **45. Sternheim et al., 2011, UK (Sternheim et al., 2011)** | What does uncertainty mean to women with anorexia nervosa? An interpretative phenomenological analysis | To explore the construct of intolerance of uncertainty in patients with anorexia nervosa through focus groups. | Inpatient, rehabilitation and day-patient eating disorders services | Female aged 15-46; 9 | Interpretive Phenomenological Analysis |
| **46. Stockford, 2018, UK (Stockford, 2018)** | Severe and enduring anorexia nervosa: the personal meaning of symptoms and treatment | Explore the experience of individuals suffering from the severe and enduring form of AN | Specialist eating disorder service (in/outpatients) | Female aged 33-48 (M 36); 6 | Semi-structured interview; Interpretive Phenomenological Analysis |
| **47. Surgenor et al., 2003, NZ (Surgenor et al., 2003)** | ‘‘Knowing One’s Self’’ Anorexic: Implications for  Therapeutic Practice | To explore how individuals with AN experience the ‘self’ within their condition | Inpatients and outpatient at specialised ED service | Female, diagnosed with AN, age unreported; 7 | Focus groups; Deconstructive approach |
| **48. Tan, Hope and Stewart, 2003, UK (Tan et al., 2003)** | Anorexia nervosa and personal identity: The accounts of patients and their parents | To attempt to understand the effects of having anorexia nervosa on the sense of personal identity of patients, particularly young women who may not have completed the developmental task of identity formation prior to developing the disorder. | Unreported | Female, aged 13-22; 10 | Self-report instrument and semi-structured interview |
| **49. Tierney & Fox, 2010, UK (Tierney & Fox, 2010)** | Living with the ‘anorexic voice’: a thematic analysis | To establish the perspectives of those with anorexia who identified with the concept of living with an anorexic voice. | Advertisement by three self-help organisations | Mean age 22.1 (SD 6.1); 21 | Thematic Analysis |
| **50. Tozzi et al., 2003, NZ (Tozzi et al., 2003)** | Causes and Recovery in Anorexia Nervosa:  The Patient’s Perspective | To assess the subjective experience of women who had a past or current diagnosis of AN. | Former patients of specialised ED service in Christchurch | Female, history of AN, mean age 32.3; 70 | Semi-structured interview |
| **51. Williams et al., 2016, UK (K. Williams et al., 2016)** | Sense of self and anorexia nervosa: a grounded theory | To explore the nature of the relationship between the self and the eating disorder in individuals with a lifetime history of AN. | 2 NHS outpatient eating disorder services & charity advertisements e.g., BEAT UK | Female aged 18-60; 11 | Semi-structured interview; Constructivist Grounded Theory Methodology |
| **52. Williams & Reid, 2010, Worldwide (S. Williams & Reid, 2010)** | Understanding the experience of ambivalence in anorexia nervosa: the maintainer’s perspective | To investigate the experiences and understandings of those who wish to maintain their anorexia and how these understandings may affect their treatment experience. | Online ‘pro-ana’ websites | Individuals aged 18-36; 13 | Online focus groups and email interviews; Interpretive Phenomenological Analysis |
| **53. Williams & Reid, 2012, Worldwide (S. Williams & Reid, 2012)** | “It’s like there are two people in my head:” a phenomenological exploration of anorexia nervosa and its relationship to the self | To explore the lived experience of AN from the perspective of those who use pro-recovery websites for eating disorders. | Online ‘pro-recovery’ websites | Individuals aged 21-50 (M 27); 14 | Email interview; Interpretive Phenomenological Analysis |

**Appendix C.** CASP quality assessment – included studies

| STUDY No. | 1 | 2 | 3 | 4 | 5 | 6 | 7 | 8 | 9 | 10 | 11 | 12 | 13 | 14 | 15 | 16 | 17 | 18 | 19 | 20 | 21 | 22 | 23 | 24 | 25 | 26 |
| --- | --- | --- | --- | --- | --- | --- | --- | --- | --- | --- | --- | --- | --- | --- | --- | --- | --- | --- | --- | --- | --- | --- | --- | --- | --- | --- |
| 1. Clear statement of aims? | 3 | 3 | 3 | 3 | 3 | 2 | 2 | 3 | 3 | 3 | 3 | 3 | 3 | 3 | 3 | 3 | 3 | 3 | 2 | 2 | 3 | 3 | 3 | 3 | 3 | 3 |
| 2. Appropriate  methodology? | 3 | 2 | 3 | 3 | 3 | 3 | 3 | 3 | 3 | 3 | 2 | 3 | 3 | 3 | 3 | 3 | 3 | 3 | 3 | 3 | 3 | 3 | 3 | 3 | 3 | 3 |
| 3. Appropriate research design? | 3 | 3 | 3 | 3 | 3 | 3 | 3 | 3 | 3 | 3 | 3 | 3 | 3 | 3 | 3 | 3 | 3 | 3 | 3 | 2 | 3 | 3 | 3 | 3 | 3 | 3 |
| 4. Appropriate recruitment strategy? | 3 | 3 | 3 | 3 | 3 | 1 | 1 | 3 | 3 | 3 | 3 | 3 | 3 | 3 | 3 | 2 | 3 | 3 | 3 | 2 | 3 | 3 | 3 | 3 | 3 | 3 |
| 5. Appropriate data collection? | 3 | 3 | 3 | 3 | 3 | 2 | 2 | 3 | 3 | 3 | 3 | 3 | 3 | 3 | 3 | 3 | 3 | 3 | 3 | 3 | 3 | 3 | 3 | 3 | 3 | 3 |
| 6. Relationship between researcher and participants considered? | 1 | 2 | 3 | 2 | 1 | 3 | 1 | 1 | 3 | 2 | 2 | 1 | 2 | 1 | 3 | 1 | 1 | 2 | 2 | 1 | 3 | 3 | 1 | 1 | 1 | 2 |
| 7. Ethical issues considered? | 2 | 3 | 1 | 3 | 1 | 3 | 1 | 2 | 3 | 3 | 2 | 2 | 2 | 1 | 2 | 1 | 1 | 1 | 3 | 2 | 3 | 1 | 1 | 1 | 1 | 3 |
| 8. Rigorous data analysis? | 3 | 3 | 2 | 3 | 2 | 1 | 2 | 2 | 3 | 3 | 3 | 3 | 3 | 3 | 3 | 3 | 3 | 3 | 3 | 3 | 3 | 3 | 3 | 3 | 3 | 3 |
| 9. Clear statement of findings? | 3 | 3 | 3 | 3 | 3 | 3 | 3 | 3 | 3 | 3 | 3 | 3 | 3 | 3 | 3 | 3 | 3 | 3 | 3 | 3 | 3 | 3 | 3 | 3 | 3 | 3 |
| 10. How valuable is the research? | 3 | 2 | 3 | 3 | 2 | 2 | 3 | 2 | 2 | 2 | 2 | 2 | 2 | 3 | 2 | 2 | 3 | 3 | 3 | 3 | 3 | 3 | 3 | 3 | 2 | 2 |
| Total | 27 | 27 | 27 | 29 | 24 | 23 | 21 | 25 | 29 | 28 | 26 | 26 | 27 | 26 | 28 | 24 | 26 | 27 | 28 | 24 | 30 | 28 | 26 | 26 | 25 | 28 |

| STUDY No. | 27 | 28 | 29 | 30 | 31 | 32 | 33 | 34 | 35 | 36 | 37 | 38 | 39 | 40 | 41 | 42 | 43 | 44 | 45 | 46 | 47 | 48 | 49 | 50 | 51 | 52 | 53 |
| --- | --- | --- | --- | --- | --- | --- | --- | --- | --- | --- | --- | --- | --- | --- | --- | --- | --- | --- | --- | --- | --- | --- | --- | --- | --- | --- | --- |
| 1. Clear statement of aims? | 3 | 3 | 3 | 3 | 3 | 3 | 3 | 3 | 3 | 3 | 3 | 3 | 2 | 3 | 3 | 3 | 3 | 3 | 3 | 3 | 1 | 1 | 3 | 3 | 3 | 3 | 1 |
| 2. Appropriate  methodology? | 3 | 3 | 3 | 3 | 2 | 3 | 3 | 3 | 3 | 2 | 3 | 3 | 3 | 3 | 3 | 3 | 3 | 3 | 3 | 3 | 3 | 3 | 3 | 3 | 3 | 3 | 3 |
| 3. Appropriate research design? | 3 | 3 | 3 | 3 | 2 | 2 | 3 | 3 | 3 | 3 | 2 | 3 | 3 | 3 | 3 | 3 | 3 | 2 | 2 | 3 | 2 | 3 | 3 | 3 | 3 | 3 | 3 |
| 4. Appropriate recruitment strategy? | 3 | 3 | 3 | 2 | 1 | 3 | 3 | 2 | 3 | 2 | 3 | 3 | 3 | 3 | 3 | 1 | 2 | 1 | 2 | 3 | 3 | 2 | 3 | 3 | 2 | 3 | 3 |
| 5. Appropriate data collection? | 3 | 3 | 3 | 1 | 1 | 3 | 3 | 3 | 3 | 3 | 2 | 3 | 3 | 3 | 3 | 2 | 3 | 3 | 3 | 3 | 2 | 3 | 3 | 3 | 2 | 3 | 1 |
| 6. Relationship between researcher and participants considered? | 1 | 1 | 1 | 1 | 1 | 1 | 1 | 2 | 3 | 1 | 3 | 2 | 2 | 1 | 3 | 1 | 2 | 3 | 1 | 3 | 1 | 1 | 2 | 1 | 3 | 1 | 3 |
| 7. Ethical issues considered? | 1 | 3 | 3 | 1 | 2 | 3 | 1 | 3 | 2 | 1 | 1 | 3 | 1 | 1 | 3 | 1 | 2 | 3 | 2 | 1 | 1 | 1 | 3 | 3 | 3 | 1 | 1 |
| 8. Rigorous data analysis? | 3 | 3 | 3 | 1 | 1 | 3 | 3 | 3 | 3 | 2 | 3 | 3 | 3 | 3 | 1 | 2 | 2 | 3 | 3 | 3 | 2 | 1 | 3 | 3 | 2 | 3 | 3 |
| 9. Clear statement of findings? | 3 | 3 | 3 | 2 | 3 | 3 | 3 | 3 | 3 | 3 | 3 | 3 | 3 | 3 | 3 | 2 | 2 | 2 | 3 | 3 | 2 | 2 | 3 | 2 | 3 | 3 | 3 |
| 10. How valuable is the research? | 3 | 3 | 3 | 1 | 2 | 3 | 3 | 3 | 3 | 3 | 3 | 3 | 3 | 3 | 1 | 2 | 1 | 3 | 3 | 3 | 2 | 3 | 3 | 3 | 3 | 3 | 2 |
| Total | 26 | 28 | 28 | 18 | 20 | 27 | 26 | 28 | 29 | 23 | 26 | 29 | 26 | 26 | 26 | 20 | 27 | 26 | 25 | 28 | 19 | 20 | 28 | 27 | 28 | 26 | 29 |

**Appendix D.** Identified references by third-order construct and sub-theme

| Third-order construct | Sub-themes | Studies |
| --- | --- | --- |
| Emotion as overwhelming  82 | Anger (5)  Loneliness/isolation (12)  Sadness (16)  Fear (26)  Shame (7)  Hopelessness (6)  Difficulty understanding or identifying emotions (10) | (Cooper et al., 2007; Espeset et al., 2012b; J. R. E. Fox, 2009; Marzola et al., 2015; Ross & Green, 2011)  (Arkell & Robinson, 2008; J. R. Fox & Diab, 2015; Freedman et al., 2006; Hannon et al., 2017; Koruth et al., 2012; Leppanen et al., 2021; Marzola et al., 2015; O’Shaughnessy et al., 2013; Rance et al., 2017; Rich, 2006; Robinson et al., 2015; Ross & Green, 2011)  (Button & Warren, 2001; Cooper et al., 2007; Dahlborg Lyckhage et al., 2015; Espeset et al., 2012a, 2012b; J. R. E. Fox, 2009; Freedman et al., 2006; Hannon et al., 2017; Koruth et al., 2012; Lavis, 2018; Petersson et al., 2021; Robinson et al., 2015; Serpell et al., 1999; Skårderud, 2007b, 2007a; S. Williams & Reid, 2010)  (Arkell & Robinson, 2008; Blackburn et al., 2020; Broomfield et al., 2021; Chan & Ma, 2003; Dahlborg Lyckhage et al., 2015; Espeset et al., 2012a; J. R. Fox & Diab, 2015; J. R. E. Fox, 2009; Godier & Park, 2015; Higbed & Fox, 2010; Hope et al., 2011; Konstantellou et al., 2019; Koruth et al., 2012; Lavis, 2018; Malson, 1999; Marzola et al., 2015; J. E. O’Connell et al., 2018; O’Shaughnessy et al., 2013; Petersson et al., 2021; Ross & Green, 2011; Skårderud, 2007a; Sternheim et al., 2011; Stockford, 2018; K. Williams et al., 2016; S. Williams & Reid, 2010)  (Conti, 2016; Espeset et al., 2012a; Koruth et al., 2012; Marzola et al., 2015; Petersson et al., 2021; Rance et al., 2017; Skårderud, 2007b)  (Arkell & Robinson, 2008; J. R. Fox & Diab, 2015; Hannon et al., 2017; Higbed & Fox, 2010; Rich, 2006; Stockford, 2018)  (Broomfield et al., 2021; Cooper et al., 2007; Cruzat‐mandich et al., 2017; Espeset et al., 2012a; J. R. E. Fox, 2009; Koruth et al., 2012; Kyriacou et al., 2009; Major et al., 2019; O’Shaughnessy et al., 2013; Petersson et al., 2021) |
| Identity  79 | Lost sense of self (11)  Role/intertwined  (19)  Achievement/  strength (22)  Perfect/pride (12)  Low self-efficacy/esteem (15) | (Broomfield et al., 2021; Conti, 2016; Higbed & Fox, 2010; Hope et al., 2011; Leppanen et al., 2021; L. O’Connell, 2021; Petersson et al., 2021; Rance et al., 2017; Ross & Green, 2011; Tan et al., 2003; K. Williams et al., 2016)  (Arkell & Robinson, 2008; Bates, 2015; Broomfield et al., 2021; Cruzat‐mandich et al., 2017; J. R. Fox & Diab, 2015; Higbed & Fox, 2010; Hope et al., 2011; Malson, 1999; Marzola et al., 2015; L. O’Connell, 2021; Rance et al., 2017; Rich, 2006; Robinson et al., 2015; Sternheim et al., 2011; Stockford, 2018; Tan et al., 2003; Tierney & Fox, 2010; K. Williams et al., 2016; S. Williams & Reid, 2010)  (Arkell & Robinson, 2008; Bates, 2015; Chan & Ma, 2003; Conti, 2016; Faija et al., 2017; A. P. Fox et al., 2011; Freedman et al., 2006; Gooldin, 2008; Granek, 2007; Hannon et al., 2017; Higbed & Fox, 2010; Malson, 1999; Marzola et al., 2015; Mulkerrin et al., 2016; Nordbø et al., 2006; Rich, 2006; Robinson et al., 2015; Skårderud, 2007b, 2007a; Tierney & Fox, 2010; Tozzi et al., 2003; S. Williams & Reid, 2010)  (Bates, 2015; Faija et al., 2017; Granek, 2007; Mulkerrin et al., 2016; L. O’Connell, 2021; Stockford, 2018; Surgenor et al., 2003; Tan et al., 2003; Tierney & Fox, 2010; Tozzi et al., 2003; S. Williams & Reid, 2010, 2012)  (Arkell & Robinson, 2008; Bates, 2015; Blackburn et al., 2020; Cooper et al., 2007; Dahlborg Lyckhage et al., 2015; Godier & Park, 2015; Major et al., 2019; Rance et al., 2017; Robinson et al., 2015; Skårderud, 2007b, 2007a; Sternheim et al., 2011; Stockford, 2018; Tierney & Fox, 2010; S. Williams & Reid, 2010) |
| Tool  76 | Guardian/  protector (24)  Avoidance/  numbing (26)  Control (23)  Becoming needless/self-punishment (3) | (Arkell & Robinson, 2008; Blackburn et al., 2020; Broomfield et al., 2021; Chan & Ma, 2003; Dahlborg Lyckhage et al., 2015; A. P. Fox et al., 2011; Freedman et al., 2006; Godier & Park, 2015; Hannon et al., 2017; Higbed & Fox, 2010; Hope et al., 2011; Konstantellou et al., 2019; Lavis, 2018; Marzola et al., 2015; Nordbø et al., 2006; J. E. O’Connell et al., 2018; Serpell et al., 1999; Skårderud, 2007a; Stockford, 2018; Surgenor et al., 2003; Tierney & Fox, 2010; K. Williams et al., 2016; S. Williams & Reid, 2010, 2012)  (Arkell & Robinson, 2008; Broomfield et al., 2021; Button & Warren, 2001; Dahlborg Lyckhage et al., 2015; Espeset et al., 2012b; A. P. Fox et al., 2011; J. R. Fox & Diab, 2015; J. R. E. Fox, 2009; Freedman et al., 2006; Godier & Park, 2015; Hannon et al., 2017; Higbed & Fox, 2010; Konstantellou et al., 2019; Kyriacou et al., 2009; Lavis, 2018; Marzola et al., 2015; Nordbø et al., 2006; Petersson et al., 2021; Robinson et al., 2015; Serpell et al., 1999; Skårderud, 2007a; Stockford, 2018; Surgenor et al., 2003; K. Williams et al., 2016; S. Williams & Reid, 2010, 2012)  (Arkell & Robinson, 2008; Broomfield et al., 2021; Button & Warren, 2001; Conti, 2016; Cruzat‐mandich et al., 2017; Dahlborg Lyckhage et al., 2015; Espeset et al., 2012b; J. R. Fox & Diab, 2015; Freedman et al., 2006; Godier & Park, 2015; Granek, 2007; Higbed & Fox, 2010; Konstantellou et al., 2019; Major et al., 2019; Marzola et al., 2015; Petersson et al., 2021; Serpell et al., 1999; Skårderud, 2007b, 2007a; Sternheim et al., 2011; Tozzi et al., 2003; S. Williams & Reid, 2010, 2012)  (Blackburn et al., 2020; Dahlborg Lyckhage et al., 2015; S. Williams & Reid, 2010) |
| Conflict  30 | In control vs out of control (12)  Wish to maintain illness vs wish to recover (9)  Functional impairment (9) | (Conti, 2016; Cruzat‐mandich et al., 2017; Dahlborg Lyckhage et al., 2015; A. P. Fox et al., 2011; J. R. Fox & Diab, 2015; Godier & Park, 2015; Lavis, 2018; Mulkerrin et al., 2016; Rance et al., 2017; Tierney & Fox, 2010; S. Williams & Reid, 2010, 2012)  (Cruzat‐mandich et al., 2017; A. P. Fox et al., 2011; J. R. Fox & Diab, 2015; Higbed & Fox, 2010; L. O’Connell, 2021; Surgenor et al., 2003; Tan et al., 2003; Tierney & Fox, 2010; S. Williams & Reid, 2010)  (Arkell & Robinson, 2008; Blackburn et al., 2020; Button & Warren, 2001; A. P. Fox et al., 2011; Freedman et al., 2006; Godier & Park, 2015; Hope et al., 2011; Robinson et al., 2015; Stockford, 2018) |
| Interpersonal communication  24 | Difficulty with interpersonal relationships (8)  Difficulty verbalising distress (10)  Need for social approval (6) | (Arkell & Robinson, 2008; Blackburn et al., 2020; Espeset et al., 2012a; J. R. Fox & Diab, 2015; Koruth et al., 2012; Kyriacou et al., 2009; Leppanen et al., 2021; O’Shaughnessy et al., 2013)  (Cruzat‐mandich et al., 2017; Dahlborg Lyckhage et al., 2015; Espeset et al., 2012b; J. R. E. Fox, 2009; Koruth et al., 2012; Kyriacou et al., 2009; Marzola et al., 2015; Nordbø et al., 2006; Serpell et al., 1999; S. Williams & Reid, 2010)  (Espeset et al., 2012a; Faija et al., 2017; Freedman et al., 2006; Granek, 2007; O’Shaughnessy et al., 2013; Sternheim et al., 2011) |
| Corporeality  20 | Disconnection from/incongruence with bodily reality (7)  Hunger experience (3)  Need to be ‘empty’ (3)  Need to disappear (7) | (Bates, 2015; Cruzat‐mandich et al., 2017; Espeset et al., 2012a; Gooldin, 2008; Higbed & Fox, 2010; Malson, 1999; Rich, 2006)  (Gooldin, 2008; Rance et al., 2017; Skårderud, 2007b)  (Cooper et al., 2007; Espeset et al., 2012a; Skårderud, 2007a)  (Bates, 2015; Cooper et al., 2007; Cruzat‐mandich et al., 2017; Malson, 1999; Nordbø et al., 2006; Skårderud, 2007b; K. Williams et al., 2016) |

**References**

Arkell, J., & Robinson, P. (2008). A pilot case series using qualitative and quantitative methods: Biological, psychological and social outcome in severe and enduring eating disorder (anorexia nervosa). *International Journal of Eating Disorders*, *41*(7), 650–656. https://doi.org/10.1002/eat.20546

Bates, C. F. (2015). “I Am a Waste of Breath, of Space, of Time”: Metaphors of Self in a Pro-Anorexia Group. *Qualitative Health Research*, *25*(2), 189–204. https://doi.org/10.1177/1049732314550004

Blackburn, B., O’Connor, J., & Parsons, H. (2020). Becoming needless: A psychoanalytically informed qualitative study exploring the interpersonal and intrapsychic experiences of longstanding anorexia nervosa. *International Journal of Applied Psychoanalytic Studies*, *n/a*(n/a), 1–15. https://doi.org/10.1002/aps.1679

Broomfield, C., Rhodes, P., & Touyz, S. (2021). How and why does the disease progress? A qualitative investigation of the transition into long-standing anorexia nervosa. *Journal of Eating Disorders*, *9*(1), 103. https://doi.org/10.1186/s40337-021-00458-w

Button, E. J., & Warren, R. L. (2001). Living with anorexia nervosa: The experience of a cohort of sufferers from anorexia nervosa 7.5 years after initial presentation to a specialized eating disorders service. *European Eating Disorders Review*, *9*(2), 74–96. https://doi.org/10.1002/erv.400

Chan, Z., & Ma, J. (2003). Anorexic Body: A Qualitative Study. *Forum Qualitative Sozialforschung / Forum: Qualitative Social Research*, *4*(1), Article 1. https://doi.org/10.17169/fqs-4.1.758

Conti, J. E. (2016). “I Don’t Think Anorexia Is the Way Out”: Reconstruction of Meaning in Women’s Narratives of Anorexia Nervosa over 10 Years. *Journal of Constructivist Psychology*, *29*(2), 165–183. https://doi.org/10.1080/10720537.2015.1072485

Cooper, M. J., Deepak, K., Grocutt, E., & Bailey, E. (2007). The experience of ‘feeling fat’ in women with anorexia nervosa, dieting and non-dieting women: An exploratory study. *European Eating Disorders Review*, *15*(5), 366–372. https://doi.org/10.1002/erv.785

Cruzat‐mandich, C., Díaz‐castrillón, F., Escobar‐koch, T., & Simpson, S. (2017). From eating identity to authentic selfhood: Identity transformation in eating disorder sufferers following psychotherapy. *Clinical Psychologist*, *21*(3), 227–235. https://doi.org/10.1111/cp.12067

Dahlborg Lyckhage, E., Gardvik, A., Karlsson, H., Törner Mulari, J., & Berndtsson, I. (2015). Young Women With Anorexia Nervosa: Writing Oneself Back Into Life. *SAGE Open*, *5*(1), 2158244015576549. https://doi.org/10.1177/2158244015576549

Espeset, E. M. S., Gulliksen, K. S., Nordbø, R. H. S., Skårderud, F., & Holte, A. (2012a). Fluctuations of Body Images in Anorexia Nervosa: Patients’ Perception of Contextual Triggers. *Clinical Psychology & Psychotherapy*, *19*(6), 518–530. https://doi.org/10.1002/cpp.760

Espeset, E. M. S., Gulliksen, K. S., Nordbø, R. H. S., Skårderud, F., & Holte, A. (2012b). The Link Between Negative Emotions and Eating Disorder Behaviour in Patients with Anorexia Nervosa. *European Eating Disorders Review*, *20*(6), 451–460. https://doi.org/10.1002/erv.2183

Faija, C. L., Tierney, S., Gooding, P. A., Peters, S., & Fox, J. R. E. (2017). The role of pride in women with anorexia nervosa: A grounded theory study. *Psychology and Psychotherapy: Theory, Research and Practice*, *90*(4), 567–585. https://doi.org/10.1111/papt.12125

Fox, A. P., Larkin, M., & Leung, N. (2011). The Personal Meaning of Eating Disorder Symptoms: An Interpretative Phenomenological Analysis. *Journal of Health Psychology*, *16*(1), 116–125. https://doi.org/10.1177/1359105310368449

Fox, J. R., & Diab, P. (2015). An exploration of the perceptions and experiences of living with chronic anorexia nervosa while an inpatient on an Eating Disorders Unit: An Interpretative Phenomenological Analysis (IPA) study. *Journal of Health Psychology*, *20*(1), 27–36. https://doi.org/10.1177/1359105313497526

Fox, J. R. E. (2009). A qualitative exploration of the perception of emotions in anorexia nervosa: A basic emotion and developmental perspective. *Clinical Psychology & Psychotherapy*, *16*(4), 276–302. https://doi.org/10.1002/cpp.631

Freedman, G., Leichner, P., Manley, R., Sandhu, P. S., & Wang, T. C. (2006). Understanding anorexia nervosa through analysis of thematic content of letters in an adolescent sample. *European Eating Disorders Review*, *14*(5), 301–307. https://doi.org/10.1002/erv.691

Godier, L. R., & Park, R. J. (2015). Does compulsive behavior in Anorexia Nervosa resemble an addiction? A qualitative investigation. *Frontiers in Psychology*, *6*. https://doi.org/10.3389/fpsyg.2015.01608

Gooldin, S. (2008). Being anorexic: Hunger, subjectivity, and embodied morality. *Medical Anthropology Quarterly*, *22*(3), 274–296. https://doi.org/10.1111/j.1548-1387.2008.00026.x

Granek, L. (2007). “You’re a Whole Lot of Person”—Understanding the Journey Through Anorexia to Recovery: A Qualitative Study. *The Humanistic Psychologist*, *35*(4), 363–385. https://doi.org/10.1080/08873260701593367

Hannon, J., Eunson, L., & Munro, C. (2017). The patient experience of illness, treatment, and change, during intensive community treatment for severe anorexia nervosa. *Eating Disorders*, *25*(4), 279–296. https://doi.org/10.1080/10640266.2017.1318626

Higbed, L., & Fox, J. R. E. (2010). Illness perceptions in anorexia nervosa: A qualitative investigation. *British Journal of Clinical Psychology*, *49*(3), 307–325. https://doi.org/10.1348/014466509X454598

Hope, T., Tan, J., Stewart, A., & Fitzpatrick, R. (2011). Anorexia Nervosa and the Language of Authenticity. *Hastings Center Report*, *41*(6), 19–29. https://doi.org/10.1002/j.1552-146X.2011.tb00153.x

Konstantellou, A., Hale, L., Sternheim, L., Simic, M., & Eisler, I. (2019). The experience of intolerance of uncertainty for young people with a restrictive eating disorder: A pilot study. *Eating and Weight Disorders - Studies on Anorexia, Bulimia and Obesity*, *24*(3), 533–540. https://doi.org/10.1007/s40519-019-00652-5

Koruth, N., Nevison, C., & Schwannauer, M. (2012). A Grounded Theory Exploration of the Onset of Anorexia in Adolescence: Exploration of Anorexia Onset in Adolescence. *European Eating Disorders Review*, *20*(4), 257–264. https://doi.org/10.1002/erv.1135

Kyriacou, O., Easter, A., & Tchanturia, K. (2009). Comparing views of patients, parents, and clinicians on emotions in anorexia: A qualitative study. *Journal of Health Psychology*, *14*(7), 843–854. https://doi.org/10.1177/1359105309340977

Lavis, A. (2018). Not Eating or Tasting Other Ways to Live: A Qualitative Analysis of ‘Living Through’ and Desiring to Maintain Anorexia. *Transcultural Psychiatry*, *55*(4), 454–474. https://doi.org/10.1177/1363461518785796

Leppanen, J., Tosunlar, L., Blackburn, R., Williams, S., Tchanturia, K., & Sedgewick, F. (2021). Critical incidents in anorexia nervosa: Perspectives of those with a lived experience. *Journal of Eating Disorders*, *9*(1), 53. https://doi.org/10.1186/s40337-021-00409-5

Major, L., Viljoen, D., & Nel, P. (2019). The experience of feeling fat for women with anorexia nervosa: An interpretative phenomenological analysis. *European Journal of Psychotherapy & Counselling*, *21*(1), 52–67. https://doi.org/10.1080/13642537.2018.1563909

Malson, H. (1999). Womæn under erasure: Anorexic bodies in postmodern context. *Journal of Community & Applied Social Psychology*, *9*(2), 137–153. https://doi.org/10.1002/(SICI)1099-1298(199903/04)9:2<137::AID-CASP506>3.0.CO;2-I

Marzola, E., Abbate-Daga, G., Gramaglia, C., Amianto, F., & Fassino, S. (2015). A qualitative investigation into anorexia nervosa: The inner perspective. *Cogent Psychology*, *2*(1), 1032493. https://doi.org/10.1080/23311908.2015.1032493

Mulkerrin, Ú., Bamford, B., & Serpell, L. (2016). How well does Anorexia Nervosa fit with personal values? An exploratory study. *Journal of Eating Disorders*, *4*(1), 20. https://doi.org/10.1186/s40337-016-0109-z

Nordbø, R. H. S., Espeset, E. M. S., Gulliksen, K. S., Skårderud, F., & Holte, A. (2006). The meaning of self-starvation: Qualitative study of patients’ perception of anorexia nervosa. *International Journal of Eating Disorders*, *39*(7), 556–564. https://doi.org/10.1002/eat.20276

O’Connell, J. E., Bendall, S., Morley, E., Huang, C., & Krug, I. (2018). Delusion-like beliefs in anorexia nervosa: An interpretative phenomenological analysis. *Clinical Psychologist*, *22*(3), 317–326. https://doi.org/10.1111/cp.12137

O’Connell, L. (2021). Being and doing anorexia nervosa: An autoethnography of diagnostic identity and performance of illness. *Journal for the Social Study of Health*, *1*, 13634593211017190. https://doi.org/10.1177/13634593211017190

O’Shaughnessy, R., Dallos, R., & Gough, A. (2013). A Narrative Study of the Lives of Women Who Experience Anorexia Nervosa. *Qualitative Research in Psychology*, *10*(1), 42–62. https://doi.org/10.1080/14780887.2011.586100

Petersson, S., Gullbing, L., & Perseius, K.-I. (2021). Just like fireworks in my brain – a Swedish interview study on experiences of emotions in female patients with eating disorders. *Journal of Eating Disorders*, *9*(1), 24. https://doi.org/10.1186/s40337-021-00371-2

Rance, N., Clarke, V., & Moller, N. (2017). The anorexia nervosa experience: Shame, Solitude and Salvation. *Counselling and Psychotherapy Research*, *17*(2), 127–136. https://doi.org/10.1002/capr.12097

Rich, E. (2006). Anorexic dis(connection): Managing anorexia as an illness and an identity. *Sociology of Health & Illness*, *28*(3), 284–305. https://doi.org/10.1111/j.1467-9566.2006.00493.x

Robinson, P. H., Kukucska, R., Guidetti, G., & Leavey, G. (2015). Severe and Enduring Anorexia Nervosa (SEED-AN): A Qualitative Study of Patients with 20+ Years of Anorexia Nervosa: SEED-AN: A Qualitative Study. *European Eating Disorders Review*, *23*(4), 318–326. https://doi.org/10.1002/erv.2367

Ross, J. A., & Green, C. (2011). Inside the experience of anorexia nervosa: A narrative thematic analysis. *Counselling and Psychotherapy Research*, *11*(2), 112–119. https://doi.org/10.1080/14733145.2010.486864

Serpell, L., Treasure, J., Teasdale, J., & Sullivan, V. (1999). Anorexia nervosa: Friend or foe? *International Journal of Eating Disorders*, *25*(2), 177–186. https://doi.org/10.1002/(SICI)1098-108X(199903)25:2<177::AID-EAT7>3.0.CO;2-D

Skårderud, F. (2007a). Eating one’s words, Part I: ‘Concretised metaphors’ and reflective function in anorexia nervosa—an interview study. *European Eating Disorders Review*, *15*(3), 163–174. https://doi.org/10.1002/erv.777

Skårderud, F. (2007b). Shame and pride in anorexia nervosa: A qualitative descriptive study. *European Eating Disorders Review*, *15*(2), 81–97. https://doi.org/10.1002/erv.774

Sternheim, L., Konstantellou, A., Startup, H., & Schmidt, U. (2011). What does uncertainty mean to women with anorexia nervosa? An interpretative phenomenological analysis. *European Eating Disorders Review*, *19*(1), 12–24. https://doi.org/10.1002/erv.1029

Stockford, C. (2018). Severe and Enduring Anorexia Nervosa_ The personal meaning of symptoms and treatment. *Women’s Studies International Forum*, 10.

Surgenor, L. J., Plumridge, E. W., & Horn, J. (2003). “Knowing one’s self” anorexic: Implications for therapeutic practice. *International Journal of Eating Disorders*, *33*(1), 22–32. https://doi.org/10.1002/eat.10117

Tan, J. O. A., Hope, T., & Stewart, A. (2003). Anorexia nervosa and personal identity: The accounts of patients and their parents. *International Journal of Law and Psychiatry*, *26*(5), 533–548. https://doi.org/10.1016/S0160-2527(03)00085-2

Tierney, S., & Fox, J. R. E. (2010). Living with the anorexic voice: A thematic analysis. *Psychology and Psychotherapy: Theory, Research and Practice*, *83*(3), 243–254. https://doi.org/10.1348/147608309X480172

Tozzi, F., Sullivan, P. F., Fear, J. L., McKenzie, J., & Bulik, C. M. (2003). Causes and recovery in anorexia nervosa: The patient’s perspective. *International Journal of Eating Disorders*, *33*(2), 143–154. https://doi.org/10.1002/eat.10120

Williams, K., King, J., & Fox, J. R. E. (2016). Sense of self and anorexia nervosa: A grounded theory. *Psychology and Psychotherapy: Theory, Research and Practice*, *89*(2), 211–228. https://doi.org/10.1111/papt.12068

Williams, S., & Reid, M. (2010). Understanding the experience of ambivalence in anorexia nervosa: The maintainer’s perspective. *Psychology & Health*, *25*(5), 551–567. https://doi.org/10.1080/08870440802617629

Williams, S., & Reid, M. (2012). ‘It’s like there are two people in my head’: A phenomenological exploration of anorexia nervosa and its relationship to the self. *Psychology & Health*, *27*(7), 798–815. https://doi.org/10.1080/08870446.2011.595488
